# ClpX-dependent regulatory pathway exacerbates *Streptococcus pyogenes* pathogenesis in diabetic skin infection

**DOI:** 10.1128/mbio.00486-26

**Published:** 2026-06-09

**Authors:** Shuiqiao Liu, Anne L. Rosen, Benjamin S. Olson, Yongke Lu, Suzanne Hickerson, Andrew L. Kau, Scott J. Hultgren, Michael G. Caparon, Wei Xu

**Affiliations:** 1 Department of Biomedical Sciences, Marshall University Joan C. Edwards School of Medicine 12354, Huntington, West Virginia, USA; 2 Department of Molecular Microbiology, Center or Women’s Infectious Disease Research, Washington University School of Medicine 12275 , St. Louis, Missouri, USA; 3 Department of Medicine, Washington University School of Medicine 12275, St. Louis, Missouri, USA; University of California, Berkeley, Berkeley, California, USA

**Keywords:** diabetes, *Streptococcus*, infection, necrotizing, disease, neutrophils, NETs, NETosis, ClpX, CDRP, SpeB

## Abstract

Diabetes is associated with impaired immune function and increased susceptibility to severe bacterial infections, yet the pathogen-encoded mechanisms that exacerbate disease in this context remain poorly defined. *Streptococcus pyogenes* (group A *Streptococcus* [GAS]) causes invasive skin and soft tissue infections that are disproportionately severe in individuals with diabetes, often accompanied by delayed healing, excessive inflammation, and polymicrobial overgrowth. Here, we investigated how the GAS ClpX-dependent regulatory pathway (CDRP), a global virulence regulator, interacts with diabetic immune dysfunction to shape infection outcomes. Using two murine models of type I diabetes, we show that diabetic mice develop more severe and persistent GAS skin infections characterized by increased bacterial burden, exaggerated inflammatory responses, impaired neutrophil recruitment, excessive neutrophil extracellular trap (NET) accumulation, and frequent polymicrobial infections. Deletion of *clpX* significantly attenuated GAS virulence in both diabetic and non-diabetic hosts; however, the pathogenic consequences of CDRP were markedly amplified in the diabetic environment. In diabetic mice, CDRP promoted sustained inflammation, altered neutrophil behavior, impaired NET clearance, and enhanced tissue damage, leading to delayed resolution of infection. Mechanistically, ClpX-dependent virulence was associated with dysregulated protease-nuclease activity, excessive NET persistence, and defective neutrophil chemotaxis within diabetic lesions. Together, these findings indicate that while ClpX is a core regulator of GAS virulence, its downstream effects are disproportionately deleterious when host immune clearance is compromised.

**IMPORTANCE:**

Diabetic patients experience disproportionately severe bacterial infections, yet the microbial mechanisms that exacerbate disease in this immunocompromised context remain incompletely understood. This study demonstrates that the *Streptococcus pyogenes* ClpX-dependent regulatory pathway, a central regulator of virulence, amplifies tissue damage and inflammatory dysfunction during diabetic skin infection. ClpX-dependent regulation exacerbates disease by intensifying neutrophil dysregulation, excessive NET accumulation, and impaired resolution of infection in an already compromised host environment. These findings underscore the importance of host-pathogen interactions in shaping infection severity and suggest that targeting pathogen regulatory pathways may be particularly effective in settings of immune dysfunction such as diabetes.

## INTRODUCTION

Diabetes mellitus (DM), a chronic metabolic disorder marked by sustained hyperglycemia, has emerged as a significant and pressing health concern, affecting approximately 11.3% of the U.S. population (1) and over 500 million people worldwide (2–5). The elevated glucose levels, characteristic of both type 1 diabetes (T1D) and type 2 (T2D) diabetes, can disrupt local tissue homeostasis, leading to microvascular dysfunction (6), hypoxia (7, 8), an altered cytokine milieu, and compromised immunity (9, 10). As a result, diabetic individuals suffer from delayed wound healing and high rates of severe invasive infections of the skin and soft tissues (SSTIs) that are a major cause of limb amputation and hospitalization (11). Prominent among these are SSTIs caused by *Streptococcus pyogenes* (group A *Streptococcus* [GAS]), for which diabetes is the single most significant risk factor for developing a severe invasive infection (12, 13). However, the molecular mechanisms by which GAS and other pathogens exploit diabetic immune vulnerability are not fully understood (14).

The metabolic changes that accompany DM can impair multiple aspects of innate immunity, particularly the function of neutrophils, an inflammatory cell that plays a critical role in host defense against GAS infection (15). DM alters neutrophil metabolism, shunting excess glucose from glycolysis to the polyol and hexosamine pathways to prevent intracellular glucose levels from becoming toxic (16, 17). As a consequence, numerous neutrophil effector functions important for defense against GAS become dysregulated. Pathways that are inhibited include recruitment, chemotaxis, phagocytosis, and the generation of intracellular reactive oxygen species (ROS), which together reduce the capacity of neutrophils to kill bacteria (17). Stimulated pathways include the production of extracellular ROS and multiple pro-inflammatory cytokines, which combine to activate subsequent neutrophils to increase and prolong inflammation (18). DM also promotes the exaggerated release of neutrophil extracellular traps (NETs) (19), which are chromatin-based structures enriched in histones and antimicrobial proteins (19). NETs are released through a PAD4-dependent pathway (20) involving histone H3 citrullination (H3Cit) (21, 22), chromatin de-condensation, and membrane rupture (23). While NETs can immobilize and kill pathogens, their formation in non-diabetic tissue is tightly regulated and context-dependent (24).

Rather than enhancing microbial clearance, diabetic NETs often persist in wounds, forming scaffolds that amplify local inflammation and tissue injury (25). Clinical studies have linked elevated NET biomarkers, including H3Cit, myeloperoxidase (MPO)–DNA complexes, and neutrophil elastase (NE) (26, 27), with delayed healing and increased amputation risk in diabetic foot ulcer (DFU) patients (28–30). Numerous studies have shown that bacterial pathogens, including *Staphylococcus aureus* (31) and *Porphyromonas gingivalis* (32), have evolved mechanisms to induce, exploit, or evade NETs, underscoring that NETosis is not a purely host-driven defense, but a dynamic host-pathogen interface shaped by microbial cues. Thus, while it is established that the diabetic environment can exacerbate infection severity, the role of NETosis and the specific factors produced by GAS that may trigger hyper-NETosis in diabetic wounds are not well defined.

It is known that GAS has evolved a suite of virulence factors that interact with and disable neutrophil effector functions, including NETosis. These include factors that impair intracellular killing by ROS, chemokine-degrading proteases that disrupt chemotaxis, capsular polysaccharides that interfere with recognition by Toll-like receptors, and surface proteins that impede opsonophagocytosis (33–38). GAS also secretes DNases, such as Sda1 (39, 40), that degrade NETs and facilitate escape from extracellular traps (41). GAS also secretes SpeB, a cysteine protease that can inactivate numerous host immune mediators (42–46) and can also inactivate the NET-degrading Sda1 DNase (47). Thus, GAS has the potential to either promote or impede the persistence of NETs in the diabetic environment depending on the status of its transcriptome and the expression of SpeB.

In this context, considerable study has shown that the GAS transcriptome is regulated in response to several external cues, including oxygen, pH, and glucose (48), which are likely altered in the diabetic environment. Our previous work identified ClpX, an ATP-dependent AAA+ chaperone/adaptor (49), as a key component of a regulatory network required for stress tolerance and virulence, which also regulates the expression and proteolytic activation of SpeB (50–52). ClpX forms a proteolytic complex with ClpP to degrade specific substrates, including the global transcriptional regulators SpxA1 and SpxA2, thereby controlling a regulon of virulence factors (49). How this ClpX-dependent regulatory pathway (CDRP) is influenced by the diabetic environment and may modulate infection severity in DM remains incompletely understood.

Here, we tested the hypothesis that CDRP amplifies GAS pathogenesis in the diabetic host by exploiting pre-existing defects in neutrophil function and inflammatory resolution. Using targeted genetic mutants, two complementary models of type 1 diabetes (streptozotocin-induced and Ins2Akita mice) and neutrophil functional assays, we examined how CDRP shapes infection outcomes in the context of diabetic immune dysfunction. We show that while CDRP contributes to virulence in non-diabetic hosts, it disproportionally exacerbates disease severity in DM through three interrelated processes: (i) enhancing tissue injury and altering the balance of NET degradation; (ii) promoting hyper-NETosis and impairing neutrophil directional migration; and (iii) reshaping the wound niche to support polymicrobial persistence and bacterial dissemination. While GAS is only one of multiple pathogens that can cause diabetic SSTI, it serves as a genetically tractable model to uncover how pathogen-encoded regulatory pathways interact with innate immune dysfunction in DM (53–55). Together, these findings define a host-pathogen interaction axis through which bacterial regulatory networks disproportionately worsen infection outcomes in diabetic hosts and highlight potential therapeutic targets for improving outcomes in high-risk populations.

## MATERIALS AND METHODS

### Bacterial strains

*S. pyogenes* HSC5 was cultured in Todd Hewitt + 1% yeast extract (THY) broth or C medium supplemented with 0.2% glucose (56), where indicated. ΔClpX (GCP688), ΔClpX::ClpX (GCP705), and ΔSpeB (GCP538) strains were constructed previously (56). Quantification of *S. pyogenes* CFUs utilized THY medium solidified by the addition of 1.4% agar (56), which were incubated at 37°C while anaerobically using a commercial atmospheric container (GasPak EZ, catalog no. BD 260001). All cultures were seeded from overnight cultures in C medium to an initial OD_600_ = 0.05, which were then incubated for the times indicated in the text.

### Diabetic mouse models

To render mice diabetic, 7-week-old male C57BL/6 (Charles River) mice were given one intraperitoneal injection of 200 mg/kg STZ (Sigma-Aldrich, cat No. S0130) or five consecutive intraperitoneal injections of 40 mg/kg STZ, to induce pancreatic islet β-cell death (57). Mice were considered diabetic after two consecutive readings of >250 mg/dL blood glucose. This method consistently resulted in 80% penetrance of diabetes in mice, with 20% considered as STZ-injected but non-diabetic controls. Male C57BL/6-Ins2^Akita^/J (Akita, strain # 003548) mice were purchased from The Jackson Laboratory. Diabetic mice received enhanced husbandry care, including more frequent cage changes, to maintain hygiene and minimize potential exposure to external pathogens or contaminants in urine and feces.

### Murine subcutaneous ulcer model of infection

Infection of mice was carried out following a well-established protocol (58). As sex does not influence infection outcomes (58), only male mice were utilized to minimize the number of mice used. Briefly, mice received a subcutaneous injection of about 10^7^ CFU of the indicated bacterial strains into the thigh, and the areas of the resulting ulcers were determined following days of infection from analysis of digital images using ImageJ (58). Mice were euthanized by CO_2_ with a flow rate of 30%–70% of the chamber’s volume per minute, followed by cervical dislocation(59). To assess bacterial burdens, infected skin, spleen, kidney, liver, and heart tissues were aseptically excised, homogenized, and serially diluted for enumeration of CFU by spot plating on selective agar (49). CFU counts were normalized based on the entire infected tissue volume. Hemoglobin (Hb) and hematocrit (HCT) levels were measured using an AimStrip HB hemoglobin kit (Cat# 78200). Blood was collected via tail vein puncture and immediately applied to the test strip, following the manufacturer’s instructions.

### Cell viability assay

Infected skin tissue was excised from the ulcer area of diabetic mice at 3 days post-infection. Tissue samples were mechanically dissociated using a GentleMACS dissociator in C Tubes (Miltenyi Biotec, 130-096-334) according to the manufacturer’s protocol (60). The resulting cell suspension was filtered through a 40 µm cell strainer, washed with PBS, and resuspended in staining buffer (PBS + 2% FBS). Cell viability was assessed by incubating the suspension with 1 µg/mL propidium iodide (PI, Thermo Fisher) for 10 min at room temperature, protected from light. Stained cells were placed on glass slides and imaged immediately using a fluorescence microscope. Representative fields were captured and quantified for PI-positive (dead) versus PI-negative (viable) cells using ImageJ software.

### Flow cytometry

To prepare single-cell suspensions, infected ulcers with minimal surrounding tissue were removed aseptically and placed in ice-cold PBS. Samples were mechanically dissociated using a GentleMACS dissociator (Miltenyi Biotec, 130-134-029) in C Tubes (Miltenyi Biotec, 130-093-237). The resulting cell suspension was filtered through a 40 µm cell strainer. Cell counts were determined by hemocytometer. Cells were first gated on single cells and then on CD45+ leukocytes. For staining, all antibodies were used at a dilution of 1:200. Single-cell suspensions were preincubated with anti-CD16/CD32 Fc Block antibody (BioXCell, BE0307) in PBS for 10 min at RT before staining with the following antibodies. Antibodies were obtained from BioLegend and included the following: FITC anti-CD45.2 (109806), BV605 anti-MHC II (107639), and PB anti-Ly6C (128014). The following anti-mouse antibodies were obtained from Tonbo Biosciences: PE anti-F4/80 (50-4801-U100), PercpCy5.5 anti-Ly6G (65-1276-U025), APC-Cy7 anti-CD11c (25-0114-U025), and PE-Cy7 anti-CD11b (60-0112-U100). Alexa Fluor 700 anti-CD3e was from Invitrogen (56-0033-82). APC anti-mCD74 was from R&D Systems (FAB7478A). Validation information for these antibodies is available on the vendor’s websites. Cells were stained for 20 min at 4°C, washed, and fixed in 4% methanol-free paraformaldehyde (Electron Microscopy Sciences) in PBS for 20 min at 4°C. Flow cytometry data were acquired on an LSR Fortessa cytometer (BD) and analyzed using FlowJo software (TreeStar, version 10.8.1). Gating strategies are depicted in Fig. S9.

### Cytokine analysis

The supernatants derived from infected skin samples of GAS-infected mice were stored at −80°C until analysis. Prior to cytokine analysis, the samples were thawed on ice and microcentrifuged at 11,000 × *g* for 10 min. The resulting supernatants were then transferred to new tubes (60). Cytokine quantification was performed using the Bio-Plex Pro Mouse Cytokine 23-plex Assay Kit from Bio-Rad Laboratories (Cat# M60009RDPD), following the manufacturer’s protocols.

### Immunofluorescent microscopy

Infected skin ulcers were fixed in 10% buffered formalin (Thermo Fisher Scientific) for 24 h and in 70% ethanol overnight at 4°C. Samples were then paraffin-embedded, sectioned, and stained with hematoxylin and eosin (H&E) by the Anatomic and Molecular Pathology Core Labs at Washington University (61). Immunofluorescent staining was performed as described previously (62). PE anti-neutrophil elastase (Bioss, bs-6982R-PE), FITC anti-SpeB (LSBio, C500985), FITC anti-*Streptococcus* Group A (Invitrogen, PA1-73056), Alexa Fluor 647 anti-Ly6G (BioLegend, 127609), rabbit anti-Histone H3 (citrulline R2 + R8 + R17) (Abcam, AB5103), and goat-anti rabbit IgG PE (Abcam, AB72465) antibodies were used. Fluorescent images were acquired using a Zeiss Axio Imager 2 microscope (Jena, Germany) equipped with a digital camera, and fluorescent intensity was measured with ImageJ software (https://imagej.net/ij/). Quantification of NETs was performed by measuring H3Cit^+^ and neutrophil elastase^+^ extracellular signal area per field, averaged across at least three non-overlapping fields per section.

### Isolation of bone marrow–derived neutrophils

Bone marrow neutrophils were isolated from mice using a single-step Percoll gradient (63, 64). Bone marrow cells were flushed from femurs and tibias using HBSS supplemented with 20 mM HEPES. Red blood cells were lysed by a brief hypotonic treatment with 0.2% sodium chloride for 30 s, followed by restoration of tonicity with 1.6% sodium chloride. The cell suspension was passed through a 40 µm cell strainer and resuspended in HBSS/HEPES buffer. Cells were then gently layered over an equal volume of 62% Percoll and centrifuged at 1,300 × *g* for 30 min at room temperature with no brake. The mononuclear cell layer was removed from the medium/Percoll interface, and neutrophils were collected from the loose pellet (“swirl”) at the bottom (65).

### *In vitro* chemotaxis assay

Bone marrow-derived neutrophils (BMDNs) were resuspended in HBSS supplemented with 2% mouse serum, 20 mM HEPES, 1 mM CaCl_2_, and 0.5 mM MgCl_2_ (66), and seeded into the central observation channel of Ibidi µ-Slides (65). Cells were allowed to adhere for 30 min in a humidified incubator at 37°C. Live bacteria (multiplicity of infection [MOI] 1:1) were added to the left-side reservoir to establish a chemotactic gradient. Neutrophil migration within the central channel was recorded using time-lapse brightfield microscopy (Zeiss Axio Observer Inverted Microscope) at 30-s intervals for 20 min in a temperature-controlled chamber at 37°C. In all, 30 motile neutrophils were manually tracked using the Manual Tracking plugin in ImageJ (NIH). Migration parameters and track plots were analyzed using the Chemotaxis and Migration Tool (Ibidi).

### *In vitro* NETosis assay

Mouse BMDNs were seeded in tissue-culture treated 96-well plates (Corning) at a density of 10^4^ cells/well in PBS supplemented with 20 mM HEPES and 1 mM CaCl_2_. Cells were infected with GAS strains at an MOI of 1:1 for 2 h at 37°C in 5% CO_2_, alongside uninfected control and PMA-treated positive controls. For quantification of NETosis, PicoGreen (Thermo Fisher Scientific) was added to the culture supernatant, and fluorescence intensity was measured using a BioTek Cytation 5 Cell Imaging Multimode Reader (excitation/emission: 488/520 nm; bandwidth: 20 nm). NET formation was further quantified by calculating the percentage of NET-positive cells, identified by the presence of visible extracellular web-like DNA structures, relative to the total number of neutrophils, averaged across at least three non-overlapping fields per well.

### *In vitro* neutrophil clustering assay

Mouse BMDNs were resuspended at a density of 8 × 10^5^ cells/mL in PBS supplemented with 20 mM HEPES, 1 mM CaCl_2_, and 0.5 mM MgCl_2_. Cells were added to 96-well plates and allowed to settle for about 10 min at room temperature. Neutrophil clustering behavior was recorded in brightfield using a BioTek Cytation 5 Cell Imaging Multimode Reader equipped with an environmental chamber maintained at 37°C for live-cell imaging. For analysis, the total number of clusters per field was manually counted, and the size of each cluster was measured using ImageJ (NIH). At least 10 non-overlapping fields were analyzed per condition.

### Two-photon intravital microscopy

Neutrophil behavior was imaged using intravital two-photon microscopy in the *In Vivo* Imaging Core at Washington University (67, 68). C57BL/6J control or STZ-induced diabetic mice (*n* = 3 per group) were infected with 10^7^ CFU of GAS (WT or ΔClpX) by toe injection. At 2 h post-infection, mice were anesthetized with isoflurane and placed on custom-built, temperature-controlled mounting platforms with the footpad immobilized for stable imaging. Imaging was performed on a custom-built two-photon microscope based on an Olympus IX81 upright platform, equipped with dual Coherent Vision II Ti:Sapphire lasers, four photomultiplier tube (PMT) detectors (bi- and multi-alkali), and a 20× water-dipping objective (XLUMPLFLN, 1.0 NA, Olympus). Excitation was set at 900 nm. Emission signals were collected through a series of dichroic filters (Semrock): SHG (<495 nm), GFP (495–540 nm), YFP (540–585 nm), and tdTomato (>585 nm). Time-lapse z-stacks of 41 consecutive 2 µm optical sections (512 × 512 pixels, 0.8 μm/pixel, 10-frame averaging per plane) were captured every 33 s over 40 time points using SlideBook software (Intelligent Imaging Innovations).

### Image analysis

Three-dimensional rendering, image processing, and neutrophil tracking were performed using Imaris software (version 9.8, Bitplane). Quantitative cell tracking data were analyzed using the Motility Lab online platform (2ptrack.net), which enables robust assessment of migration parameters (69).

### DNA extraction of mouse specimens, V4 16S rRNA sequencing, and analysis

Genomic DNA from mouse fecal pellets (three pellets pooled per group) and skin samples were extracted using phenol:chloroform:isoamyl alcohol and bead beating with zirconium beads (70, 71). A portion of the aqueous phase was then purified using QIAquick PCR Purification Kit (Qiagen, catalog #28106), quantified by measuring 260/280 nm absorbance, and diluted to a concentration of 5 ng/μL. Then, 10 ng of DNA was used to amplify the V4 16S rRNA region with barcoded primers as previously described (72). PCR products were quantified with Quant-iT dsDNA Broad Range Kit (Invitrogen, catalog #Q33130), pooled to equal concentration, and purified with AMPure XP SPRI beads. The final library was diluted and sequenced on an Illumina MiSeq with 2 × 250 bp chemistry. FastQ files were demultiplexed and binned into amplicon sequence variants (ASVs) using DADA2 and a custom database as previously described (70, 73). Further analysis and visualization of relative abundances were carried out using phyloseq (v1.38.0).

### Statistical analyses

Data are derived from at least three independent experiments and are presented as mean ± SEM. Statistical significance between two groups was evaluated using either a two-tailed unpaired Student’s t-test (for normally distributed data with equal variance) (74) or the two-tailed Mann–Whitney *U* test (for non-parametric comparisons) (75), as determined by the Shapiro–Wilk test for normality (76). For all experiments involving more than two groups (e.g., comparisons across diabetic/non-diabetic, WT/ΔClpX conditions), one-way or two-way ANOVA with Tukey’s *post hoc* test for multiple comparisons was applied. All statistical analyses were performed using GraphPad Prism version 10. Statistical significance is denoted as *P* < 0.05 (*), *P* < 0.01 (**), and *P* < 0.001 (***). For all analyses, the null hypothesis was rejected at *P* < 0.05. No data were excluded unless otherwise specified.

## RESULTS

### CDRP exacerbates mortality and tissue pathology in diabetic infections

**Fig 1:**
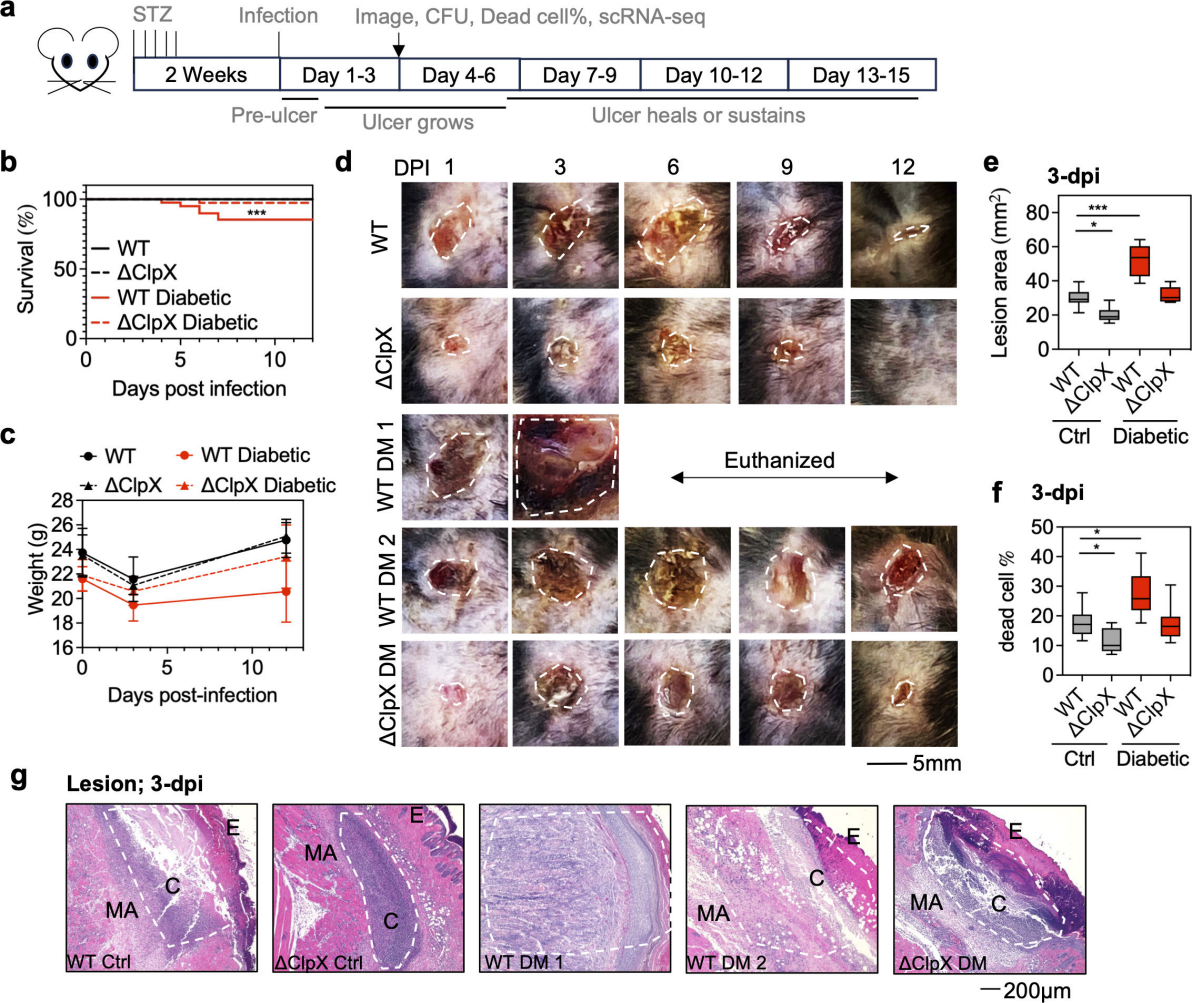
CDRP exacerbates disease severity in STZ-induced diabetic mice. (**a**) Schematic of the experimental timeline for subcutaneous ulcer infection. The arrow represents the time point for data collection. (**b and c**) Survival (**b**) and body weight change (**c**) over 12 days in control and STZ-induced diabetic C57BL/6J mice infected with 10^7^ CFU of WT or ΔClpX GAS. (**d**) Representative ulcer images with lesion areas outlined by dashed white lines. (**e**) Quantification of lesion area at 3 dpi; mean ± SEM is shown (*n* = 10 mice per group). Statistics: two-way ANOVA with Tukey’s *post hoc* test. (**f**) Percentage of dead cells among total nucleated cells from infected skin tissue, as determined by PI-positive staining. (**g**) H&E staining of infected skin sections. Infection zones are outlined by dashed white lines. DM 1: diabetic mouse 1. DM 2: diabetic mouse 2. Labels are as follows: C, core; E, eschar; MA, margin. *: *P* < 0.05, ***: *P* < 0.001.

T1D was induced in C57BL/6 mice using streptozotocin (STZ) (see Materials and Methods). Diabetic status was confirmed by sustained hyperglycemia (>250 mg/dL), while STZ-injected mice with lower glucose levels served as non-diabetic controls (Fig. S1a). Two weeks after STZ injection, diabetic mice exhibited significant weight loss and a marked reduction in subcutaneous adipose tissue compared to controls (Fig. S1b and c). To assess the role of CDRP in infection severity, we utilized a previously established murine model of subcutaneous ulcer infection (experimental timeline, Fig. 1a). At the dose used (1 × 10^7^ CFU), diabetic mice infected with WT GAS exhibited significant mortality (15% within 7 dpi) and progressive weight loss, whereas no mortality was observed in non-diabetic controls infected with WT or in diabetic mice infected with the ΔClpX mutant (Fig. 1b and c). Notably, diabetic mice infected with ΔClpX displayed a weight-change trajectory and survival rate comparable to those of non-diabetic controls, despite their lower baseline weight due to diabetes (Fig. 1c). Consistent with our previous findings (49), WT-infected ulcers were significantly larger and had higher bacterial burden than ΔClpX-infected counterparts by 3 dpi in both host backgrounds. However, the difference in disease severity between WT and ΔClpX infection was markedly amplified in diabetic mice. In non-diabetic mice, WT infection caused ulcers of about 30 mm^2^ by 6 dpi, which resolved by 12 dpi (Fig. 1d). In contrast, diabetic mice infected with WT GAS developed severe, heterogeneous ulcer phenotypes: some displayed extensive necrotic lesions requiring euthanasia by 4 dpi (WT DM 1), while others developed persistent, non-healing ulcers (WT DM 2) (Fig. 1d). On average, lesions in WT-infected diabetic mice reached about 55 mm^2^ by 3 dpi, nearly twice the size of those in non-diabetic mice and significantly larger than ΔClpX-infected diabetic mice (Fig. 1e*, P* < 0.001 for WT-DM vs. ΔClpX-DM). ΔClpX-infected ulcers were significantly smaller, with lesion areas averaging about 20 mm^2^ in non-diabetic and about 30 mm^2^ in diabetic mice (Fig. 1e). This differential severity was not attributable to STZ exposure alone, as STZ-injected mice that did not develop sustained hyperglycemia (<250 mg/dL) exhibited lesion sizes comparable to untreated controls (Fig. S2a). These findings indicate that exacerbated disease severity is associated with sustained hyperglycemia rather than STZ treatment *per se*. Live/dead staining with PI performed on dissociated cells from infected tissue revealed extensive host cell death in WT-infected diabetic mice, whereas ΔClpX-infected tissue showed more viable cells (Fig. 1f). H&E staining further demonstrated that WT infection in diabetic mice caused either full-thickness dermal necrosis (WT DM 1) or severe tissue injury with diminished immune infiltration (WT DM 2), while ΔClpX-infected skin maintained epithelial and subcutaneous integrity and displayed a greater immune cell presence (Fig. 1g).

### CDRP drives polymicrobial emergence and systemic dissemination

**Fig 2:**
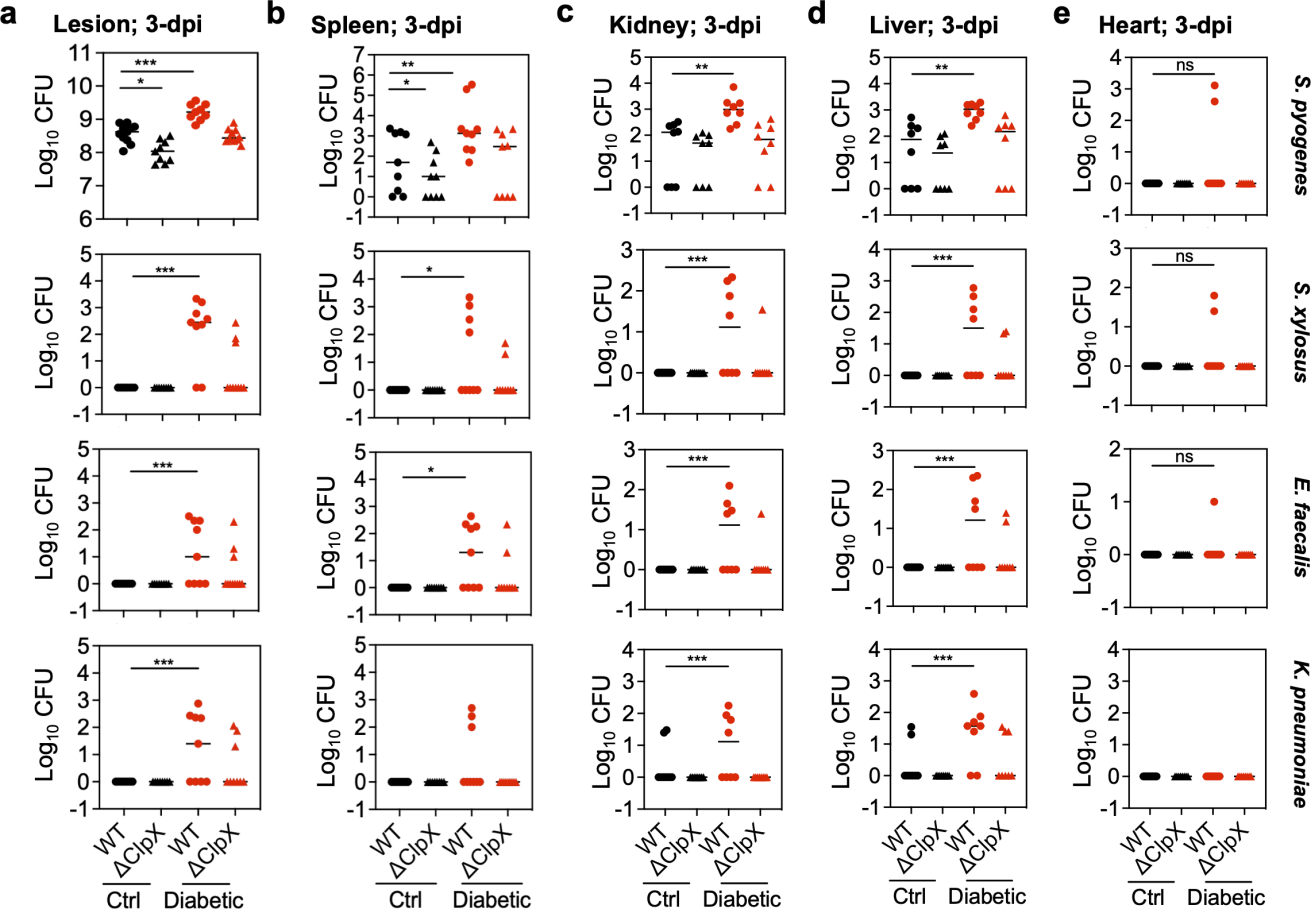
CDRP promotes polymicrobial emergence and systemic dissemination in diabetic mice. C57BL/6J control or STZ-induced diabetic mice were subcutaneously infected with 10^7^ CFU of WT or ΔClpX GAS. (**a**) Bacterial burden at the infection site at 3 dpi, showing the number of recoverable CFUs of GAS, *Staphylococcus xylosus*, *Enterococcus faecalis*, and *Klebsiella pneumoniae*. (**b–e**) Dissemination of bacteria to the spleen (**b**), kidney (**c**), liver (**d**), and heart (**e**) at 3 dpi. Each data point represents an individual mouse; data pooled from two independent experiments. ns: not significant. *: *P* < 0.05, **: *P* < 0.01, ***: *P* < 0.001 by two-way ANOVA with Tukey’s *post hoc* test.

At 3 dpi, diabetic mice infected with WT GAS harbored significantly higher bacterial loads at the ulcer site, approximately 0.5 log greater than those in non-diabetic controls, while ΔClpX-infected diabetic mice exhibited bacterial burdens 0.5 log higher than in non-diabetic animals (Fig. 2a; Fig. S2b). Strikingly, polymicrobial infections emerged in 100% of WT-infected diabetic mice but were rarely detected in non-diabetic mice or in ΔClpX-infected diabetic mice (Fig. 2a). Using selective and differential agar plating followed by 16S rRNA sequencing, we identified three predominant co-infecting species: *Staphylococcus xylosus* (about 10^2^ CFU), *Enterococcus faecalis* (about 10^1^ CFU), and *Klebsiella pneumoniae* (about 10^1^ CFU) (Fig. 2a; Fig. S2c through e; Fig. S3). These species were all detectable in the feces of both diabetic and non-diabetic mice (Fig. S4), suggesting a gut or oral origin. Additional low-abundance organisms (*Bacillus* spp., *E. coli*, and *Enterobacter* spp.) were occasionally recovered as minor constituents, though co-infections typically involved three predominant species indicated above (not shown). Culture-independent identification by V4 16S sequencing did not identify any additional non-culturable species colonizing ulcers (Fig. S4). Infection with the ΔClpX strain markedly reduced both the frequency and magnitude of polymicrobial emergence at the infection site in diabetic mice (Fig. 2a). STZ-injected non-diabetic mice had minimal polymicrobial colonization, comparable to untreated controls, confirming that the observed dysbiosis and secondary colonization were driven by diabetes rather than off-target effects of STZ (Fig. S2c through e).

Higher GAS burden at the skin site was associated with systemic dissemination in diabetic mice. At 3 dpi, WT-infected diabetic animals exhibited significantly elevated GAS CFUs in the spleen, liver, and kidney, but not in the heart, compared to non-diabetic controls (Fig. 2b through e). Notably, the co-infecting species (*S. xylosus*, *E. faecalis*, and *K. pneumoniae*) were detected in the spleen, liver, and kidney of nearly half of WT-infected diabetic mice, yet were absent from corresponding organs in non-diabetic animals (Fig. 2b through e). In contrast, diabetic mice infected with the ΔClpX strain showed markedly reduced systemic spread of both GAS and the polymicrobial community. Notably, hematological parameters, including hemoglobin concentration and hematocrit percentage, remained consistent across all infected C57BL/6J groups (diabetic/non-diabetic, WT/ΔClpX-infected), indicating comparable baseline physiology independent of metabolic or infection status (Fig. S5).

### Validation in a genetic model of diabetes

To validate these findings, we utilized Akita mice, a genetic model of T1D (77). At 3 dpi, WT-infected Akita mice exhibited significant weight loss and larger ulcers relative to ΔClpX-infection, which developed only mild lesions and modest weight change (Fig. S6a through c). Furthermore, WT infection in Akita mice resulted in polymicrobial emergence and increased dissemination to the spleen, recapitulating the key features observed in STZ-induced diabetic mice (Fig. S6d and e). Interestingly, *Staphylococcus aureus* replaced *E. faecalis* as the dominant co-infecting species in Akita mice, possibly reflecting strain-specific differences in gut microbiota composition (Fig. S6d). Together, these results indicate that while CDRP contributes to GAS virulence in both host backgrounds, its impact on mortality, tissue damage, and disease persistence is disproportionately exacerbated in the context of T1D.

### SpeB mediates virulence in diabetes

CDRP is a known positive regulator of the cysteine protease SpeB, promoting both its expression and proteolytic activation; deletion of *clpX* leads to a marked reduction in SpeB activity (78–81). To directly assess the contribution of SpeB to the exacerbated disease severity observed in the diabetic context, we compared infections using a ClpX-complemented strain (ΔClpX::ClpX) and a SpeB-deficient mutant (ΔSpeB) alongside WT and ΔClpX strains in both control and diabetic mice. As expected, the ΔClpX::ClpX strain restored virulence to WT levels, recapitulating the severe pathology observed with WT infection. In diabetic mice, this included pronounced tissue destruction, elevated GAS burden at the infection site, increased bacterial dissemination to the spleen, and the re-emergence of polymicrobial co-infection (Fig. S7). Direct comparison confirmed that ΔClpX::ClpX was not significantly different from WT GAS in key diabetic outcomes (lesion size, CFU burden), confirming that the attenuated phenotype of ΔClpX is due to loss of ClpX function. In contrast, infection with the ΔSpeB strain resulted in markedly attenuated disease. Diabetic mice infected with ΔSpeB exhibited limited tissue damage, reduced GAS burden at the skin site, minimal dissemination to the spleen, and a low frequency of polymicrobial colonization, phenotypes that closely resemble those seen with ΔClpX infection and are significantly different from WT infection in diabetic hosts. These results demonstrate that SpeB, a key effector downstream of CDRP (49), mediates the majority of amplified virulence of GAS in diabetic mice, providing a mechanistic link between the ClpX regulatory pathway and detrimental host outcomes.

### CDRP modulates cytokine signaling and immune cell recruitment in diabetic hosts

**Fig 3:**
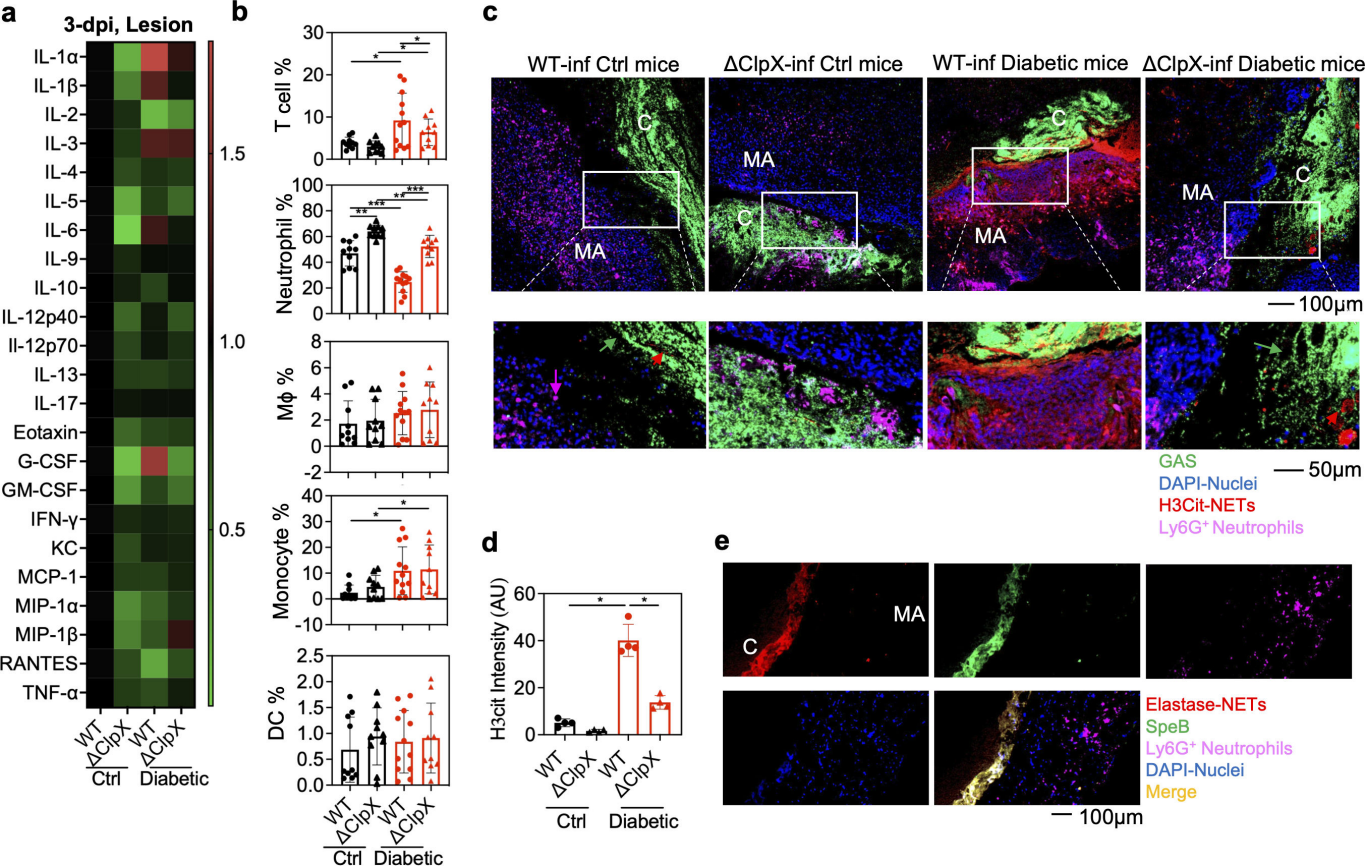
CDRP promotes systemic inflammation and neutrophil NETosis in diabetic skin infection. C57BL/6J control or STZ-induced diabetic mice were subcutaneously infected with 10^7^ CFU of WT or ΔClpX GAS. (**a**) Cytokine levels in supernatants of infected tissue at 3 dpi. Heatmap depicts fold change relative to the average level in WT-infected non-diabetic controls (set as 1). See Fig. S8 for absolute values. (**b**) Flow cytometric analysis of immune cell populations at 3 dpi. The relative abundance of indicated cell types was quantified within the total CD45+ population, isolated from the ulcer with minimal surrounding tissue. DCs, dendritic cells. Each point represents an individual mouse (*n* = 8–12 per group); data pooled from two independent experiments. (**c**), Representative fluorescent microscopy images of infected skin sections at 3 dpi, stained with anti-Ly6G (Alexa Fluor 647), anti-*S*. *pyogenes* (FITC), anti-H3Cit (PE), and DAPI. Green arrows, bacteria; red arrows, NETs; purple arrows, neutrophils. Lower panels show magnified views of boxed regions. (**d**) H3Cit+ fluorescence intensity was quantified in tissue sections using ImageJ. AU: artificial unit. (**e**), Representative fluorescent microscopy images of WT-infected diabetic skin sections stained with anti-Ly6G (Alexa Fluor 647), anti-SpeB (FITC), anti-neutrophil elastase (PE), and DAPI. C, Core; MA, margin. Data represent mean ± SEM. *: *P* < 0.05, **: *P* < 0.01, ***: *P* < 0.001 by two-way ANOVA with Tukey’s *post hoc* test.

To dissect how CDRP shapes host–pathogen interactions in diabetic infection, we first assessed cytokine responses in GAS-infected diabetic and non-diabetic mice. WT-infected diabetic mice exhibited sustained elevations in multiple neutrophil-associated and inflammatory cytokines, including IL-1α, IL-1β, IL-6, and granulocyte colony-stimulating factor (G-CSF), accompanied by suppression of the anti-inflammatory cytokine IL-10 and the chemokine RANTES (Fig. 3a; Fig. S8). By contrast, infection with ΔClpX GAS resulted in significantly lower levels of IL-5, G-CSF, GM-CSF, IL-12p40, and IL-12p70 irrespective of diabetic status, suggesting that CDRP modulates the magnitude and composition of the cytokine response, though we note that differences in local bacterial burden may contribute to this profile. Notably, IL-2 was consistently decreased, and IL-3 increased in diabetic mice regardless of the infecting strain, indicating diabetes-specific modulation of select cytokines. To further delineate host immune responses, we analyzed immune cell populations in infected tissue at 3 dpi using flow cytometry (gating strategy in Fig. S9). All immune cell populations were quantified as a percentage of CD45^+^ cells. Diabetic mice exhibited a 1.5-fold increase in CD45^+^CD11b^+^F4/80^+^ macrophages and CD45^+^CD11b^+^Ly6G^−^Ly6C^+^ monocytes compared to non-diabetic controls, independent of GAS strain. Neutrophils (CD11b^+^Ly6G^+^) constituted the dominant immune population; however, their abundance was markedly reduced in diabetic mice. Specifically, WT-infected diabetic mice showed a 2-fold reduction in neutrophils relative to non-diabetic mice, while ΔClpX-infected diabetic mice displayed a 1.5-fold reduction. In addition, neutrophil counts in ΔClpX-infected diabetic mice were approximately twofold higher than in their WT-infected counterparts (Fig. 3b). CD45^+^CD11b^−^CD3e^+^ T cells were more abundant in WT-infected diabetic mice than in ΔClpX-infected ones. Dendritic cell numbers were unchanged across all groups. These data indicate that diabetes alters the immune landscape at the infection site and that CDRP further modulates this environment, particularly by restricting neutrophil accumulation during infection.

### CDRP amplifies NET formation in diabetes

Given the known contribution of neutrophils to GAS pathogenesis (82), we next assessed NET formation in infected tissue using citrullinated histone H3 (H3Cit) as a NET marker. WT-infected diabetic mice exhibited extensive H3Cit-positive structures, consistent with exuberant NET formation, which was markedly reduced in non-diabetic mice and in ΔClpX-infected diabetic mice (Fig. 3c; Fig. S10). NETs partially co-localized with Ly6G^+^ neutrophils, but a substantial H3Cit signal was also observed in Ly6G^-^ regions, consistent with prior observations (19, 83) of neutrophil degranulation and NET detachment (Fig. S11). Quantification revealed a fivefold increase in NET formation in WT-infected diabetic mice relative to non-diabetic controls, and a threefold increase compared to ΔClpX-infected diabetic mice (Fig. 3d), indicating that CDRP-driven hyper-NETosis is potentiated by diabetic conditions. These findings were corroborated using neutrophil elastase (NE) as an independent NET marker (Fig. S12 and S13). Strikingly, SpeB signal co-localized with NE-positive NET structures in WT-infected diabetic tissue (Fig. 3e), raising the possibility that extracellular SpeB associates with NETs and leverages them as a scaffold to concentrate proteolytic activity, thereby exacerbating tissue damage. While the precise molecular mechanisms remain to be elucidated, these data collectively indicate that CDRP amplifies NETosis in diabetic skin infection, potentially through SpeB-dependent or SpeB-associated mechanisms.

### Diabetes impairs early neutrophil recruitment, while CDRP disrupts directed neutrophil migration

**Fig 4:**
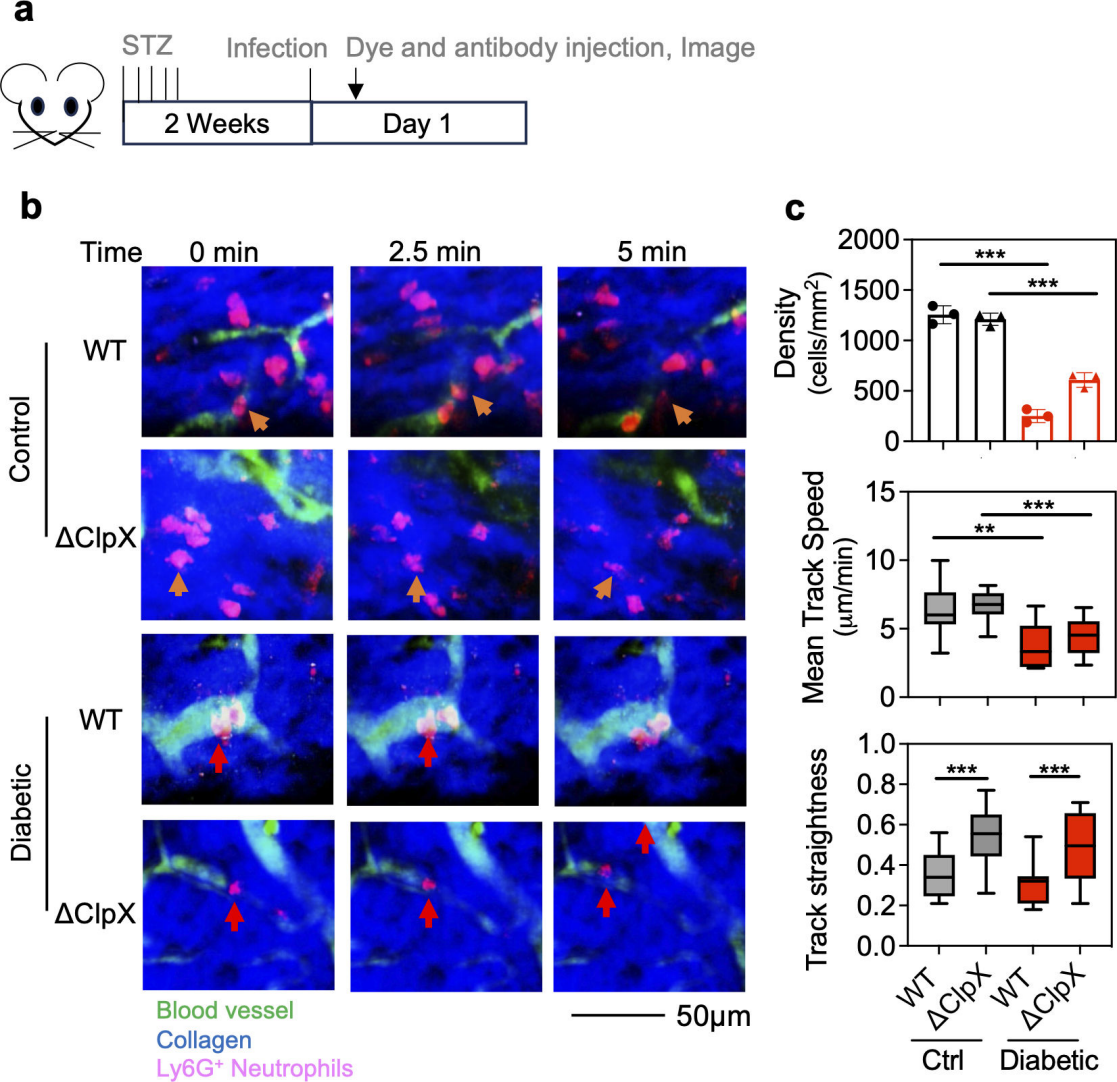
Diabetes impairs early neutrophil recruitment, while CDRP disrupts directed neutrophil migration. C57BL/6J control or STZ-induced diabetic mice were infected in the toe with 10^7^ CFU of WT or ΔClpX GAS, followed 1 h later by intravenous injection of Alexa Fluor 647 anti-Ly6G (neutrophils) and FITC-dextran (vasculature). (**a**) Schematic of the experimental design. The arrow represents the time point for data collection. (**b**) Representative time-lapse intravital two-photon microscopy (2P-IVM) images showing early neutrophil migration at 2 hpi. Arrows indicate Ly6G+ neutrophils. Blue signal denotes second harmonic generation (SHG) from collagen fibers. (**c**) Quantification of neutrophil responses. Top: neutrophil density at 2.5 min. Middle: mean track speed. Bottom: track straightness. Tracks from 100 individual cells (*n* = 3 mice per group) were manually traced in FIJI. Data are pooled from two independent experiments. Bars denote mean ± SEM. *: *P* < 0.05, **: *P* < 0.01, ***: *P* < 0.001 by two-way ANOVA with Tukey’s *post hoc* test.

To investigate how CDRP influences the spatial organization of the early neutrophil response, we employed a toe-pad infection model coupled with intravital two-photon microscopy (2P-IVM). Mice were infected with 10^7^ CFU of WT or ΔClpX GAS into the toe pad, followed 2 h later by intravenous injection of anti-Ly6G fluorescent antibody to label neutrophils and FITC-dextran to visualize vasculature (Fig. 4a). Consistent with known diabetic neutrophil dysfunction, recruitment was globally impaired in diabetic mice regardless of the infecting GAS strain. In non-diabetic mice, neutrophils responded rapidly and robustly to both WT and ΔClpX infection, infiltrating infection sites with >threefold higher density (cells/mm^2^) than in diabetic mice (Fig. 4b and c). In contrast, diabetic mice exhibited markedly impaired recruitment regardless of strain, with neutrophils migrating more slowly and occupying lesions with delay (Fig. 4c). Despite this global impairment in diabetic hosts, loss of CDRP partially restored directional precision: neutrophils in ΔClpX-infected diabetic mice migrated with 2.1-fold straighter tracks and 1.2-fold higher velocity compared to those in WT-infected diabetic mice (Fig. 4c). Thus, while diabetes reduces the magnitude and speed of early neutrophil infiltration, CDRP specifically disrupts directional sensing and spatial coordination at the infection site in diabetic hosts.

### CDRP impairs neutrophil chemotaxis and drives NETosis *in vitro*

**Fig 5:**
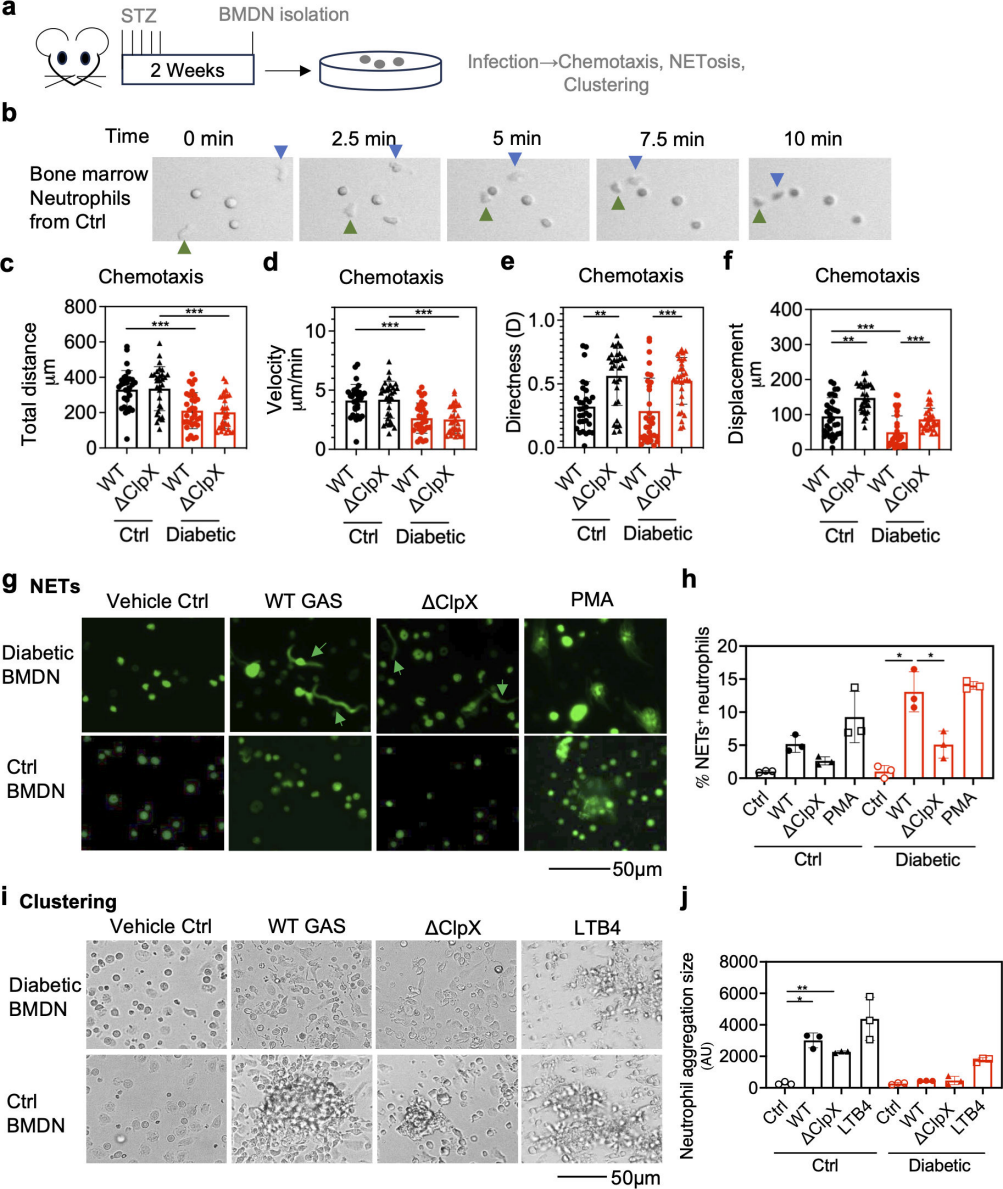
CDRP impairs neutrophil chemotaxis and drives NETosis *in vitro*. BMDNs from control or STZ-induced diabetic C57BL/6J mice were isolated using Percoll gradient centrifugation. (**a**) Schematic of *in vitro* infection and imaging workflow. (**b**) Representative time-lapse DIC images show neutrophil migration tracks in response to WT GAS (MOI 1:1) using neutrophils from non-diabetic mice. (**c–f**) Quantification of chemotaxis parameters using the Ibidi Chemotaxis and Migration Tool: total distance (**c**), displacement (**d**), velocity (**e**), and directness (**f**). Each dot represents a single neutrophil. Data pooled from two independent experiments. (**g**) Representative images of NETs formation by PicoGreen fluorescence after 2 h of exposure to GAS (MOI 1:1). (**h**) NETs were quantified by measuring PicoGreen fluorescence localized to web-like extracellular DNA structures. (**i**) Representative DIC image of neutrophil clusters. (**j**) Quantification of cluster size per field. Bars represent mean ± SEM. *: *P* < 0.05, **: *P* < 0.01, ***: *P* < 0.001 by two-way ANOVA with Tukey’s *post hoc* test.

To determine the effects of CDRP on neutrophil function, we isolated bone marrow-derived neutrophils (BMDNs) from diabetic and non-diabetic mice and performed *in vitro* assays of chemotaxis, NETosis, and cell clustering (experimental design, Fig. 5a). Neutrophil purity exceeded 91%, as determined by Ly6G^+^ staining (Fig. S14), with minimal contamination from debris or other cell types.

Neutrophil chemotaxis was assessed using a microfluidic ibidi μ-slide assay (65) in which live GAS were loaded into one chamber and neutrophils seeded centrally (MOI 1:1). Time-lapse microscopy revealed that BMDNs responded to chemo-attractants by transitioning from a rounded morphology to a polarized, spindle-like shape, migrating toward the GAS source (Fig. 5b). Diabetic neutrophils exhibited a baseline chemotactic defect, displaying shorter total travel distances and lower average velocities, independent of the bacterial strain (Fig. 5c and d). Notably, chemotactic directionality (directness) was severely impaired in neutrophils responding to WT GAS but preserved when exposed to ΔClpX GAS, with the poorest displacement observed in diabetic BMDNs exposed to WT GAS (Fig. 5e and f).

We next evaluated NET formation using a PicoGreen-based fluorescence assay to detect extracellular DNA (84). As expected, phorbol 12-myristate 13-acetate (PMA) induced extensive NETs in both diabetic and non-diabetic BMDNs, producing characteristic web-like filamentous DNA structures (Fig. 5g). Diabetic BMDNs exhibited a heightened propensity for NET formation. Both WT GAS and ΔClpX triggered NET release, but the magnitude differed: WT GAS induced extensive DNA filaments, often with multiple projections per cell, whereas ΔClpX typically triggered only a single filament per neutrophil (green arrows, Fig. 5g). Quantitative analysis revealed that WT GAS stimulated NETosis in approximately 12% of diabetic neutrophils, approaching levels seen with PMA, while ΔClpX induced NETosis in only about 5% of diabetic BMDNs (Fig. 5h). In contrast, NET formation in non-diabetic neutrophils remained low across all conditions, underscoring the heightened NETotic responsiveness of diabetic neutrophils to GAS-derived stimuli.

Finally, we examined neutrophil clustering, a behavior associated with activation and chemotactic signaling. Clustering was more prominent in non-diabetic neutrophils under all conditions (Fig. 5i). Leukotriene B4 (LTB4) served as a positive control and induced strong clustering responses in non-diabetic BMDNs. Both WT and ΔClpX GAS supernatants triggered clustering in non-diabetic but not diabetic BMDNs (Fig. 5j), further suggesting that neutrophil responsiveness is compromised in diabetes and differentially modulated by CDRP-dependent bacterial factors. Collectively, these *in vitro* assays demonstrate that CDRP exacerbates key neutrophil dysfunctions, particularly in the context of diabetic neutrophils, mirroring and providing mechanistic support for our *in vivo* observations.

### Diabetes sustains long-term infection, but CDRP deletion mitigates bacterial persistence and inflammation

**Fig 6:**
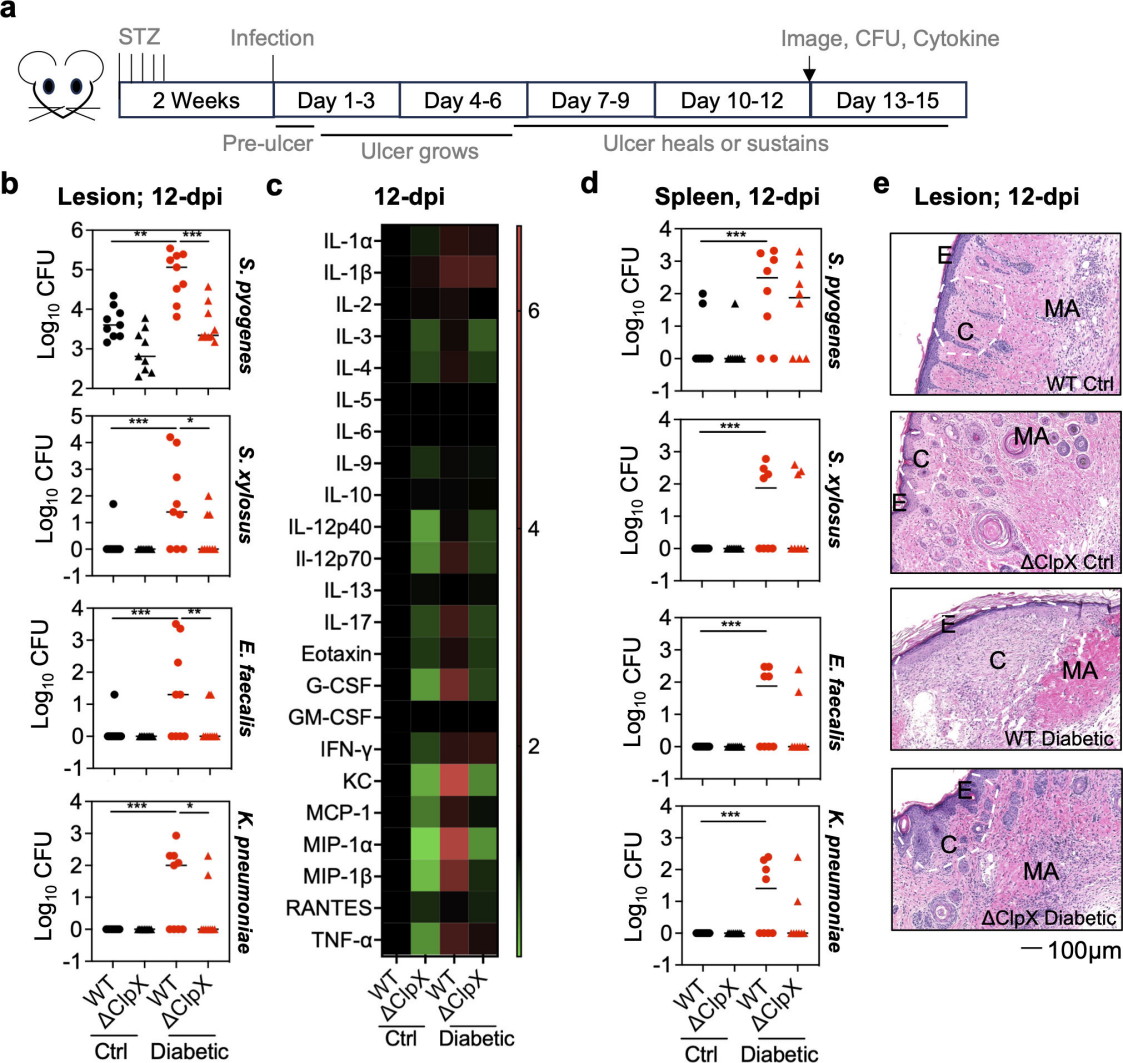
Long-term infection depends on the diabetic condition, but ClpX deletion mitigates disease severity. C57BL/6J control or STZ-induced diabetic mice were subcutaneously infected with 10^7^ CFU of WT or ΔClpX GAS. (**a**) Schematic overview of the long-term infection model. The arrow represents the time point for data collection. (**b**) Bacterial burden at the infection site at 12 dpi, showing recoverable CFUs of GAS, *Staphylococcus xylosus*, *Enterococcus faecalis*, and *Klebsiella pneumoniae*. (**c**) Cytokine levels in supernatants of infected tissue at 12 dpi. (**d**) Bacterial dissemination into the spleen at 12 dpi. (**e**) H&E-stained sections of infected skin at 12 dpi. Infection zones are outlined with dashed white lines. Labels: C, core; E, eschar; MA, margin. Each dot represents an individual mouse. Data are pooled from two independent experiments. Bars represent mean ± SEM. *: *P* < 0.05, **: *P* < 0.01, ***: *P* < 0.001 by two-way ANOVA with Tukey’s *post hoc* test.

To assess the role of CDRP in chronic infection, we extended our analysis to 12 dpi (experimental timeline, Fig. 6a). While non-diabetic mice cleared the infection by this time point, diabetic mice maintained significantly higher bacterial burdens. In diabetic mice, WT GAS persisted at the infection site with bacterial loads averaging about 10^5^ CFU, consistent with impaired immune clearance. In contrast, the ΔClpX mutant exhibited significantly lower skin burdens (about 10^3^ CFU), indicating that CDRP contributes to long-term persistence in the diabetic host (Fig. 6b). Notably, polymicrobial communities, including *Staphylococcus xylosus*, *Enterococcus faecalis*, and *Klebsiella pneumoniae*, were consistently detected in WT-infected diabetic wounds but were largely absent in ΔClpX-infected tissue, suggesting that CDRP influences the maintenance or recruitment of co-infecting species in chronic wounds.

At this late time point, cytokine levels were generally diminished compared to the acute phase; however, inflammation persisted in WT-infected diabetic skin, with elevated expression of G-CSF, keratinocyte chemoattractant (KC), MIP-1α, and IL-1β relative to WT during the acute phase (Fig. 6c; Fig. S15). ΔClpX infection induced markedly lower levels of pro-inflammatory cytokines, including G-CSF, KC, IL-12p40, and MIP-1α. Interestingly, TNF-α and MIP-1β remained relatively elevated in ΔClpX-infected diabetic mice compared to non-diabetic ones, which may reflect underlying diabetic inflammation independent of bacterial burden.

Dissemination was also assessed by quantifying bacterial burdens in the spleen. By 12 dpi, non-diabetic mice had nearly cleared the infection, with undetectable or minimal CFU. In contrast, diabetic mice harbored persistent *S. pyogenes* in the spleen (about 10^2^ CFU), highlighting a diabetes-associated defect in systemic bacterial clearance that is exacerbated by CDRP activity (Fig. 6d). Moreover, WT-infected diabetic spleens often contained polymicrobial colonizers, including *S. xylosus*, *E. faecalis*, and *K. pneumoniae*, whereas spleens from ΔClpX-infected mice were uniformly sterile.

Histological examination of infected skin revealed near-complete healing in non-diabetic mice by 12 dpi. In contrast, WT-infected diabetic wounds exhibited ongoing necrosis and disrupted tissue architecture. ΔClpX-infected diabetic wounds displayed markedly improved healing with minimal necrosis, indicating that CDRP exacerbates tissue pathology and impairs resolution during chronic infection in the diabetic host (Fig. 6e).

## DISCUSSION

In this study, we identify the CDRP as a key amplifier of GAS virulence, exacerbating infection severity in diabetic hosts. We demonstrate that CDRP worsens outcomes in T1D mice by amplifying tissue necrosis, impairing neutrophil recruitment and directionality, and triggering dysregulated NETosis. Crucially, CDRP is associated with enhanced polymicrobial co-infections and systemic dissemination, phenotypes that were markedly reduced in non-diabetic hosts or during infection with the ΔClpX mutant. Together, these findings support a model in which a bacterial regulatory pathway amplifies disease severity by interacting with host metabolic disease, accelerating tissue damage. Previous work has shown that CDRP, acting through SpxA1/A2 and other substrates, controls multiple virulence-associated traits, including SpeB expression, resistance to host cationic defensins, stress tolerance, and metabolic adaptation (49). Our findings extend these observations by demonstrating that in a diabetic host environment, this broad regulatory capacity translates into significantly worse tissue destruction and immune evasion.

Diabetic individuals exhibit an elevated predisposition to severe bacterial infections (85). In STZ-induced T1D mice, WT GAS infection led to 15% mortality, progressive weight loss, and chronic, non-healing ulcers, whereas ΔClpX infection resulted in markedly improved outcomes, with reduced inflammation and pathology that more closely resembled non-diabetic infection courses. These differences were not attributable to hyperglycemia alone, but rather to immune dysfunction that is amplified by CDRP. Notably, CDRP enhanced tissue destruction by inducing hyper-NETosis and driving SpeB-mediated proteolysis. SpeB strongly co-localized with NE-positive NETs, suggesting an association between NET structures and localized protease activity, whereby NETs may serve as structural scaffolds that retain proteases at sites of inflammation, prolonging their local activity and amplifying tissue damage. Since CDRP directly influences SpeB production, these results suggest that the CDRP-SpeB axis represents a plausible mechanism by which GAS leverages NET structures to maximize proteolytic damage in susceptible hosts. This mechanism mirrors observations in *Staphylococcus aureus* infection, where proteases degrade extracellular matrix and promote inflammation (86). Our work extends this concept to GAS, revealing a pathogen-driven cycle of NET-mediated protease retention and tissue injury that is potentiated in diabetes.

Mechanistically, CDRP impairs neutrophil recruitment and function. Flow cytometry profiling revealed significant alterations in immune cell populations, particularly neutrophils and macrophages, in CDRP-dependent and diabetes-specific contexts. Neutrophil recruitment was impaired in diabetic mice, as confirmed by intravital imaging and reduced Ly6G^+^ infiltration at infected sites. CDRP further sabotaged neutrophil function by disrupting chemotactic directness: WT GAS disrupted the directional migration of diabetic neutrophils, a defect that was restored by the ΔClpX mutant. These findings suggest that CDRP may modulate secreted effectors that interfere with chemokine sensing, thereby compounding the neutropenic state and limiting effective bacterial clearance. Impairments in neutrophil chemotaxis and migration have been observed with other pathogens that express leukotoxins or interfere with G protein-coupled receptors (GPCR) signaling, including *Porphyromonas gingivalis* (87, 88), *Escherichia coli* (89), and *Pseudomonas aeruginosa* (90). Whether CDRP regulates such factors directly or indirectly remains to be elucidated, but our data suggest it functions as a regulatory hub that coordinates immune evasion.

*In vitro* assays confirmed that CDRP promotes excessive NET formation in the context of diabetic neutrophils. WT GAS induced high-frequency extracellular DNA structures, while ΔClpX triggered less NET formation. Importantly, NETs and tissue destruction were tightly correlated *in vivo*, particularly in diabetic skin. These observations suggest that CDRP may co-opt the NETosis pathway not only to evade immune clearance but also to potentiate tissue injury by anchoring SpeB at sites of inflammation. While higher bacterial burden in WT-infected diabetic wounds likely amplifies NET accumulation, our *in vitro* data confirm that CDRP promotes NETosis in diabetic neutrophils independent of bacterial load. The mechanism linking ClpX to hyper-NETosis is likely indirect, arising from its broad role in proteostasis and virulence regulation, particularly through its control of SpeB expression and activity. SpeB can remodel host chemokine gradients and neutrophil signaling, thereby promoting dysregulated NET release (91). In addition, CDRP influences resistance to antimicrobial peptides and turnover of surface proteins, which may amplify neutrophil activation and ROS production (92–95). Together, these regulatory effects provide a plausible framework by which CDRP enhances hyper-NETosis in the diabetic host.

A key insight from this study is that CDRP enables GAS to reprogram the infection niche. WT infection in diabetic mice led to the emergence of polymicrobial consortia that originated from the gut and disseminated to infected wounds and systemic organs. This was significantly reduced in ΔClpX-infected diabetic mice, where co-colonization was reduced by over 50%. These findings support a model in which CDRP disrupts local immune control or epithelial barrier function, creating an ecological niche permissive to opportunistic pathogens. We note that increased bacterial burden and tissue damage likely contribute to this phenotype, consistent with the complex relationship between host immunity and microbial ecology. This observation also implies that the diabetic condition creates a unique niche fostering the growth and interaction of multiple bacterial species, thus complicating infection dynamics (96, 97). Polymicrobial diabetic foot infections are well documented clinically, often involving *S. aureus*, *P. aeruginosa*, *Streptococcus* spp., and *Enterobacteriaceae*, among others (98). Our findings provide evidence that a defined streptococcal regulatory system can enhance this complex microbial ecology in diabetes. Whether GAS directly promotes the growth of co-infecting species (e.g., through nutrient liberation or metabolic cross-feeding) or whether the polymicrobial emergence is purely a consequence of immune breakdown remains an open question. Future studies employing defined co-culture systems and gnotobiotic diabetic mice will be needed to dissect these potential synergistic interactions.

At later stages (12 dpi), WT GAS persisted in diabetic wounds at >10^5^ CFU, accompanied by sustained cytokine production (G-CSF, IL-1β, KC) and non-resolving inflammation. In contrast, ΔClpX was rapidly cleared and failed to maintain local or systemic inflammation, suggesting that CDRP is required for sustained infection and inflammation in the diabetic host. Collectively, these results establish a pathogenic axis whereby CDRP activates SpeB, triggers aberrant neutrophil responses, and modifies the infection environment to sustain inflammation and polymicrobial overgrowth under diabetic conditions.

This work positions CDRP as a virulence amplifier that potentiates GAS pathogenicity in diabetes through interconnected mechanisms: (i) SpeB activation → tissue destruction and NET scaffolding; (ii) neutrophil dysregulation → impaired clearance and hyper-NETosis; and (iii) niche modification → enhanced polymicrobial emergence. This mechanism parallels the role of ClpXP proteases in other pathogens—including *S. aureus*, *Listeria monocytogenes*, and *Salmonella*—where *clpXP* deletion compromises virulence, survival, and stress tolerance (92–95, 99–101). These findings have significant clinical implications, particularly in understanding the elevated risk of necrotizing fasciitis and chronic ulcers in diabetic patients.

Key unresolved questions remain: How does CDRP influence neutrophil chemotaxis, directly or via secreted signals? Does SpeB interact with components of NETs to increase its proteolytic function? Does polymicrobial emergence reflect barrier breakdown or immune misfiring triggered by CDRP activity? Does deletion of *sda1* (encoding the NET-degrading DNase) phenocopy the hyper-NETosis and virulence of WT GAS in diabetic mice? Future studies using NETosis-deficient models (e.g., PAD4^−/−^) and neutrophil adoptive transfer approaches will be valuable in further elucidating these mechanisms. Addressing these questions will require detailed proteomic and *in vivo* imaging approaches.

Here, we propose a model in which CDRP acts as a molecular hub that integrates pathogen proteostasis with host immunometabolic vulnerability. In diabetic hosts, CDRP activity exacerbates neutrophil dysfunction, promotes SpeB-driven tissue damage, and supports polymicrobial colonization, ultimately converting an acute infection into a chronic, non-healing wound. Targeting CDRP or downstream effectors such as SpeB activity or NET dysregulation may offer precision interventions to mitigate severe streptococcal infections in high-risk populations.

## Data Availability

All data will be made available upon reasonable request.

## SUPPLEMENTAL MATERIAL

Supplemental figures mbio.00486-26-s0001.pdf Figures S1 to S15.

ASM does not own the copyrights to Supplemental Material that may be linked to, or accessed through, an article. The authors have granted ASM a non-exclusive, world-wide license to publish the Supplemental Material files. Please contact the corresponding author directly for reuse.
