# Supplementary material for: ClpX-dependent regulatory pathway exacerbates Streptococcus pyogenes pathogenesis in diabetic skin infection
Source: mBio. 2026 Jun 9;17(7):e00486-26. doi: 10.1128/mbio.00486-26 (PMC13344041; doi:10.1128/mbio.00486-26)
Supplement: Supplemental figures — Figures S1 to S15. [file mbio.00486-26-s0001.pdf]

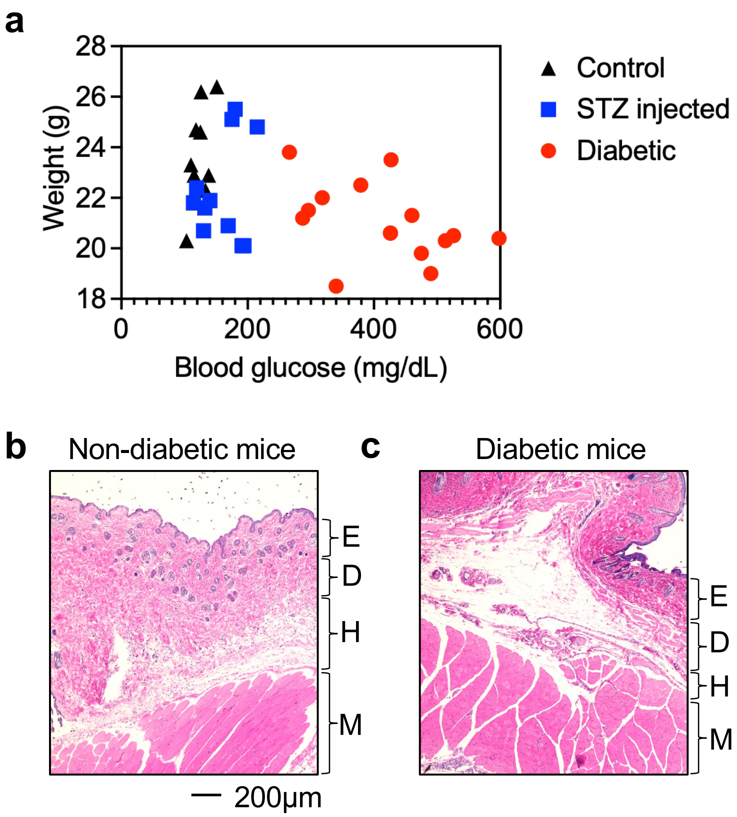

Supplementary Figure 1. Confirmation of STZ-induced type 1 diabetes in mice. C57BL/6J male mice were intraperitoneally injected with streptozotocin (STZ). Mice were classified as diabetic following two consecutive blood glucose measurements exceeding 250 mg/dL. (**a**) Correlation between body weight and blood glucose levels; each symbol represents an individual mouse. (**b**, **c**) Representative hematoxylin and eosin (H&E)-stained sections of uninfected skin from non-diabetic (**b**) and STZ-induced diabetic (**c**) mice. Tissue layers are labeled: E, epidermis; D, dermis; H, hypodermis; M, muscle.

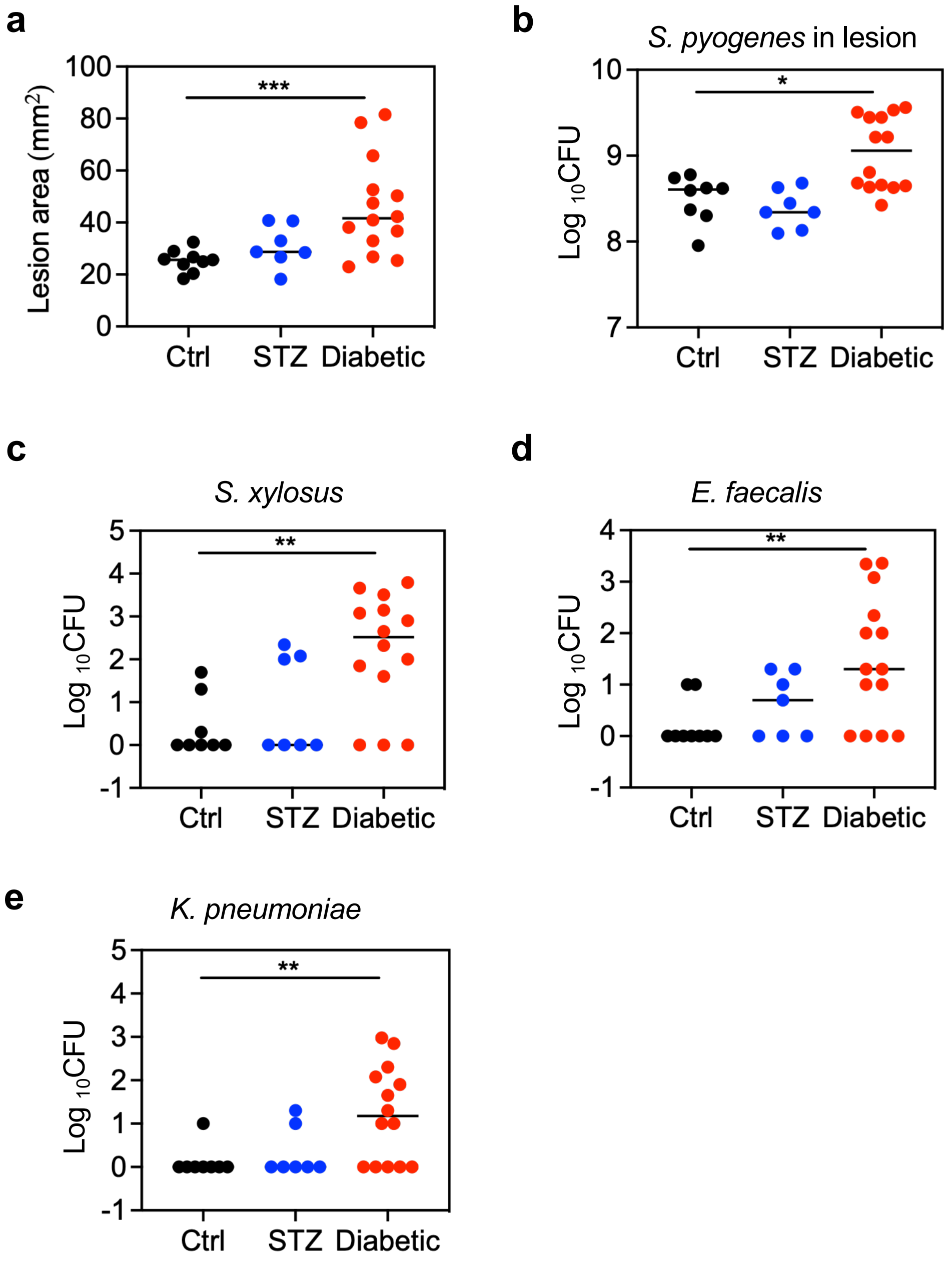

Supplementary Figure 2. Severe disease outcome in STZ-induced diabetic mice. C57BL/6J control, STZ-injected non-diabetic, and STZ-induced diabetic mice were subcutaneously infected with 10^7 CFU of WT *S. pyogenes*. (**a**) Lesion area at 3 dpi. (**b**) *S. pyogenes* burden in infected tissue. (**c**–**e**) Polymicrobial emergence in the lesion: (**c**) *Staphylococcus xylosus*, (**d**) *Enterococcus faecalis*, and (**e**) *Klebsiella pneumoniae*. Each data point represents an individual mouse. Data are pooled from two independent experiments. *, $p < 0.05$; **, $p < 0.01$; ***, $p < 0.001$ by unpaired two-tailed t-test.

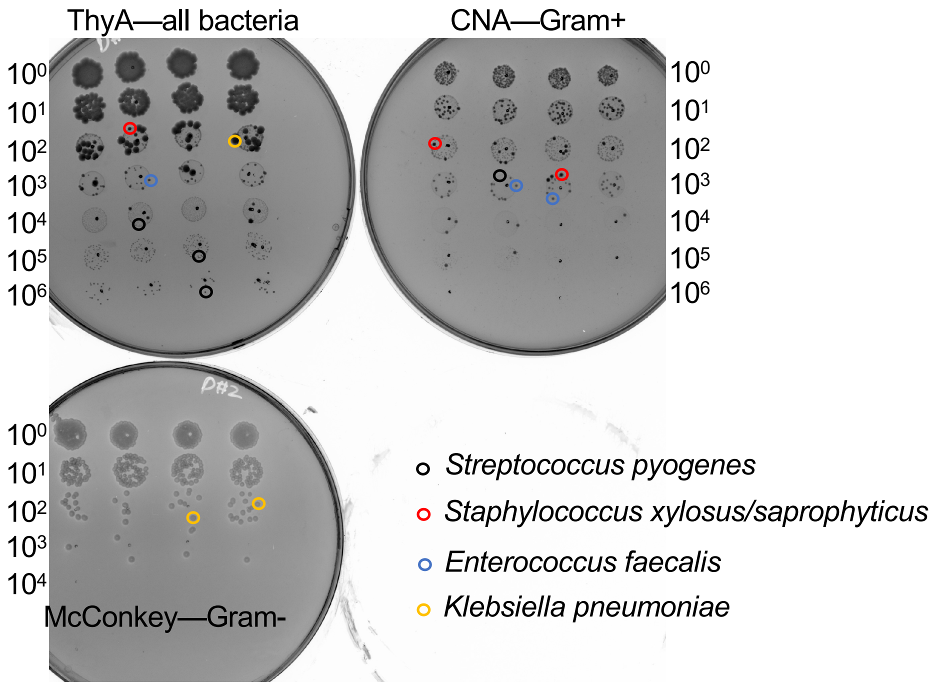

Supplementary Figure 3. Emergence of polymicrobial infection in diabetic mice. STZ-induced diabetic mice were subcutaneously infected with 10^7 CFU of WT *S. pyogenes*. At 3 dpi, infected tissue was excised, homogenized, and plated on selective and differential media including THY A, CNA (selective for Gram-positive bacteria), and MacConkey agar (selective for Gram-negative bacteria). Representative culture plates are shown. Colonies marked with circles were isolated and identified by 16S rRNA gene sequencing.

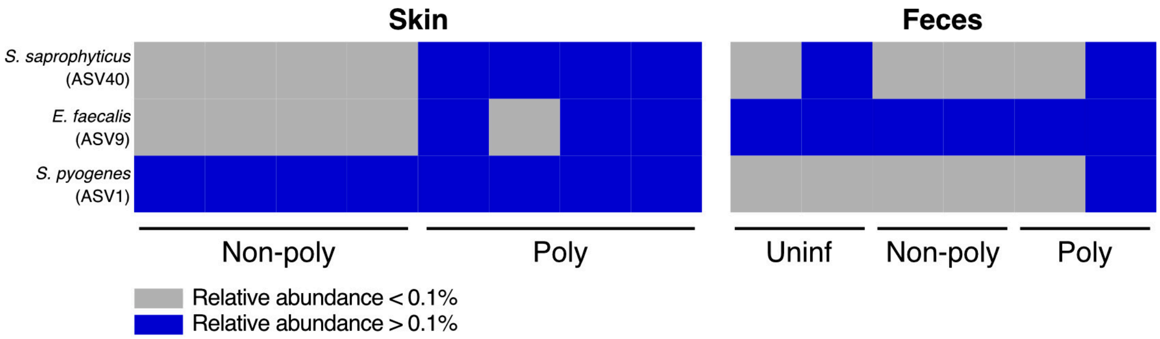

Supplementary Figure 4. Emergence of polymicrobial infection in diabetic mice. STZ-induced diabetic mice were subcutaneously infected with 10^7 CFU of WT *S. pyogenes*. At 3 dpi, infected tissue was homogenized, and fecal samples were collected. Bacterial isolates from both sources were identified by 16S rRNA gene sequencing.

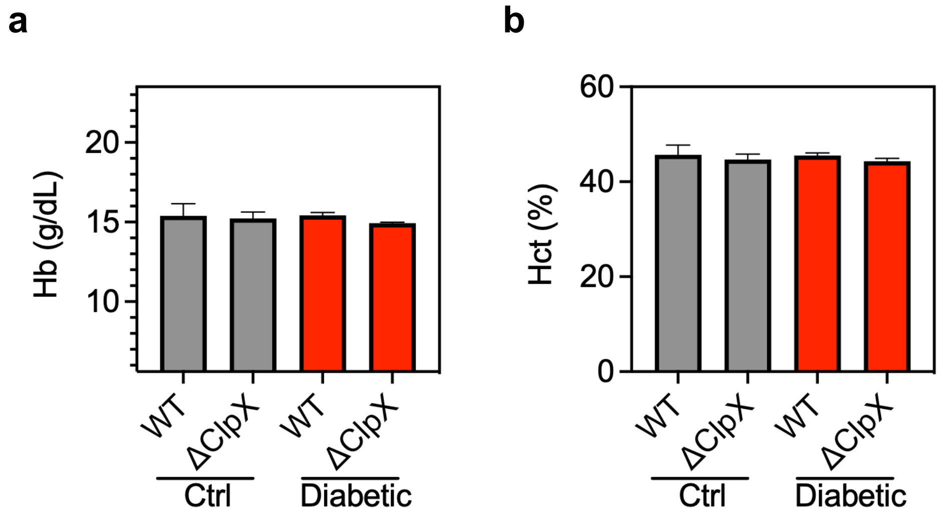

Supplementary Figure 5. Comparable hematological parameters in control and diabetic mice. C57BL/6J control and STZ-induced diabetic mice were subcutaneously infected with 10^7 CFU of W *S. pyogenes*. At 3 dpi, hemoglobin concentration (**a**) and hematocrit percentage (**b**) were measured from tail vein blood using a portable hemoglobin meter (Ermaine Laboratories, Inc.).

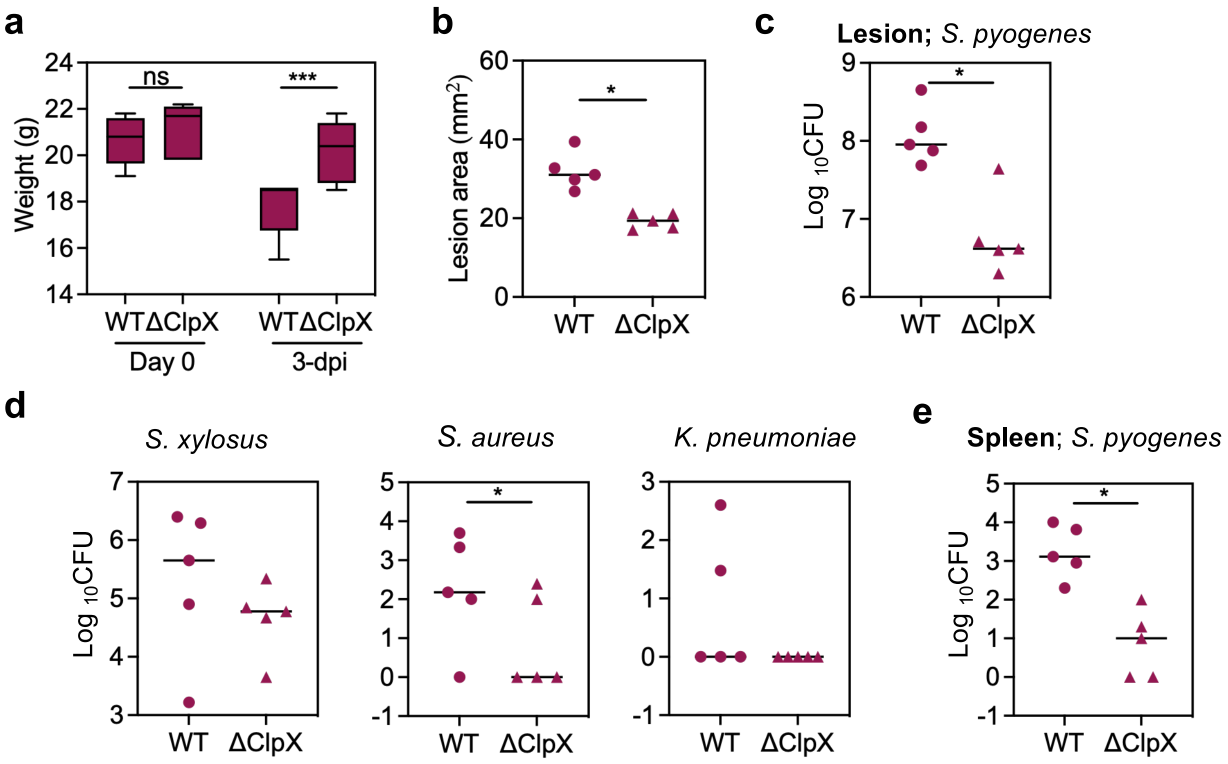

Supplementary Figure 6. ClpX promotes disease severity in Akita mice. Type 1 diabetic (Akita) mice were subcutaneously infected with 10^7 CFU of either WT or ΔClpX *S. pyogenes*. Mice were assessed for (**a**) change in body weight, (**b**) lesion area, (**c**) *S. pyogenes* burden in infected tissue, (**d**) total bacterial burden from polymicrobial emergence in infected tissue, and (**e**) *S. pyogenes* burden in the spleen. Each symbol represents an individual mouse. Data are pooled from two independent experiments. *, $p < 0.05$; ***, $p < 0.001$; ns, not significant; unpaired two-tailed t-test.

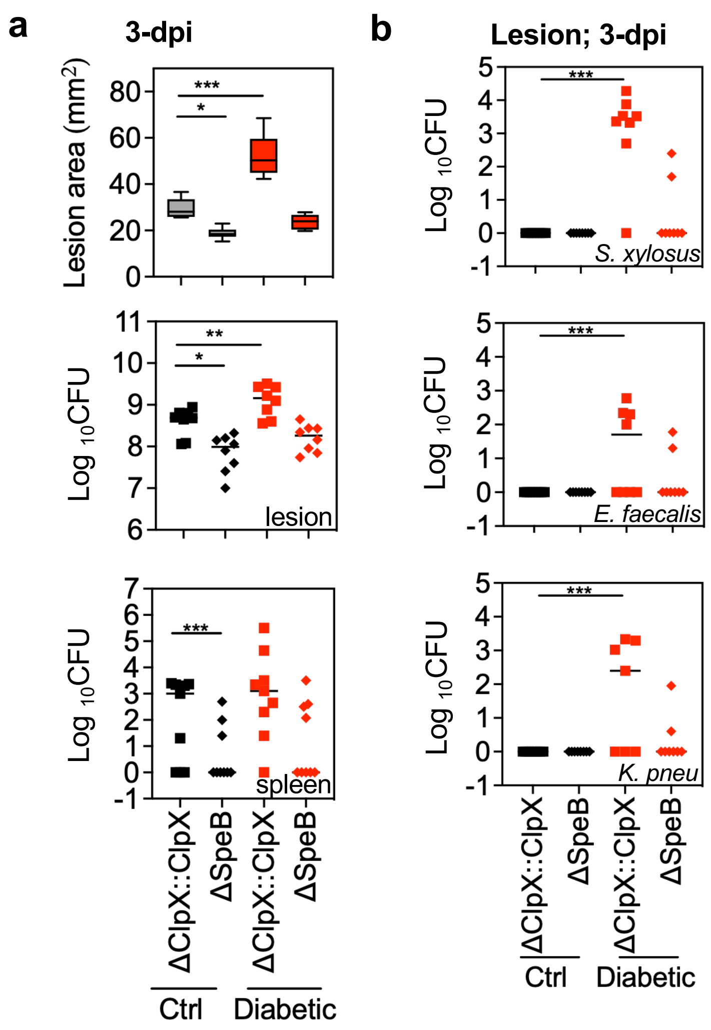

Supplementary Figure 7. SpeB is required for severe disease outcome during diabetic skin infection. C57BL/6J control or STZ-induced diabetic mice were subcutaneously infected with 10^7 CFU of either ΔClpX::ClpX or ΔSpeB strains. Lesion area (**a**-top panel), *S. pyogenes* bacterial burden in infected tissue (**a**-mid panel) or spleen (**a**-bottom panel) was assessed at 3-dpi. (**b**) Emergence of *S. xylosus*, *E. faecalis* and *K. pneumoniae* in lesion was measured at 3-dpi. *, $p < 0.05$; **, $p < 0.01$; ***, $p < 0.001$ by two-way ANOVA with Tukey's post-hoc test.

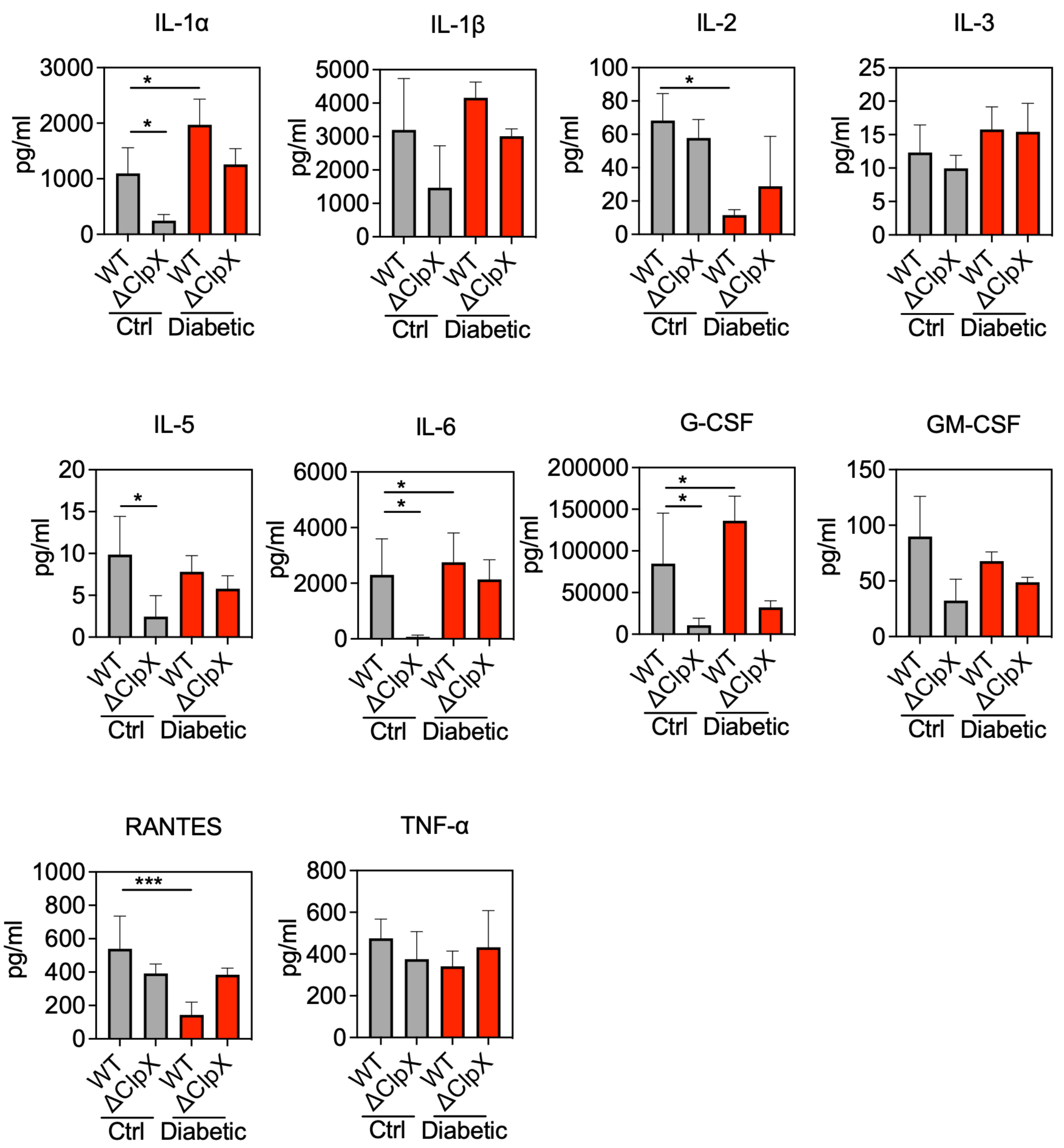

Supplementary Figure 8. ClpX promotes systemic inflammation in diabetic skin infection. C57BL/6J control and STZ-induced diabetic mice were subcutaneously infected with 10^7 CFU of WT or ΔClpX *S. pyogenes*. At 3 dpi, cytokine levels were measured in the supernatants of homogenized infected skin tissue. Data represent mean ± SEM from two independent experiments. $p < 0.05$ (*), $p < 0.001$ (***), by two-way ANOVA with Tukey's post-hoc test.

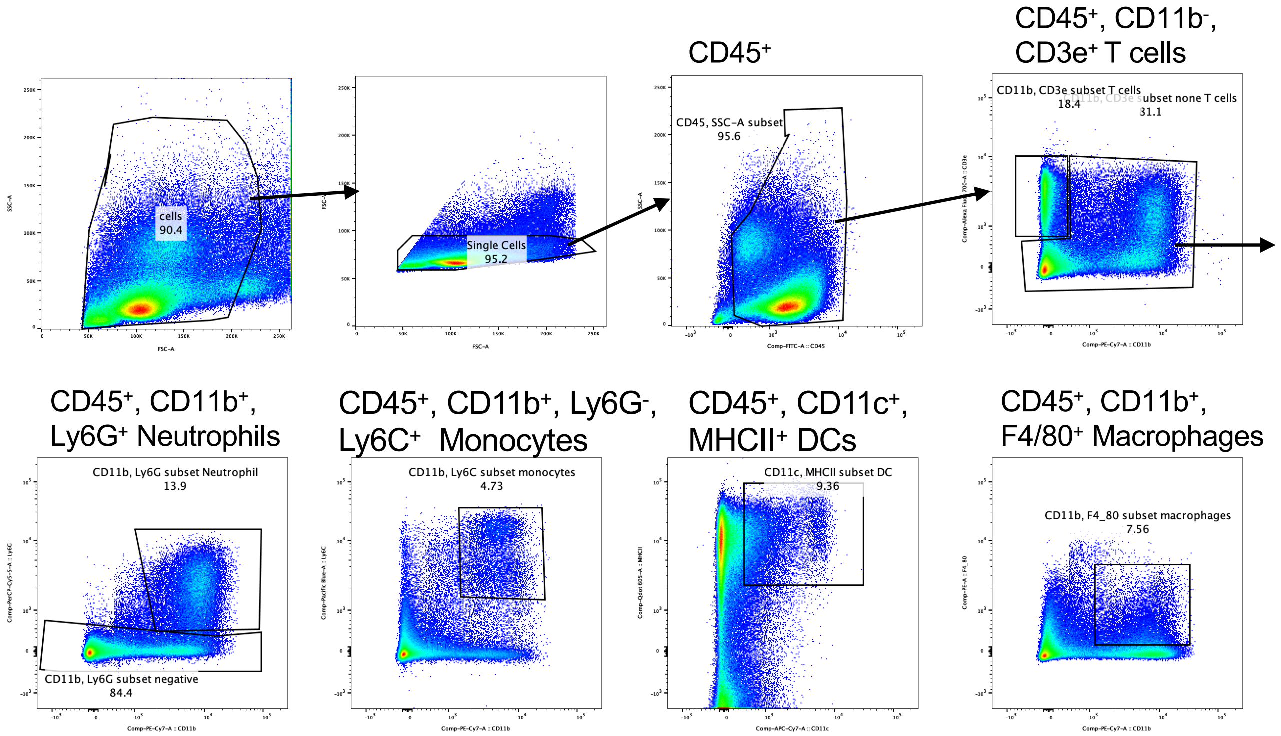

Supplementary Figure 9. FACS configuration for immune cell population. Representative flow cytometry plots for WT *Streptococcus pyogenes* infected tissue at 3 dpi, showing the gating strategy for cell populations.

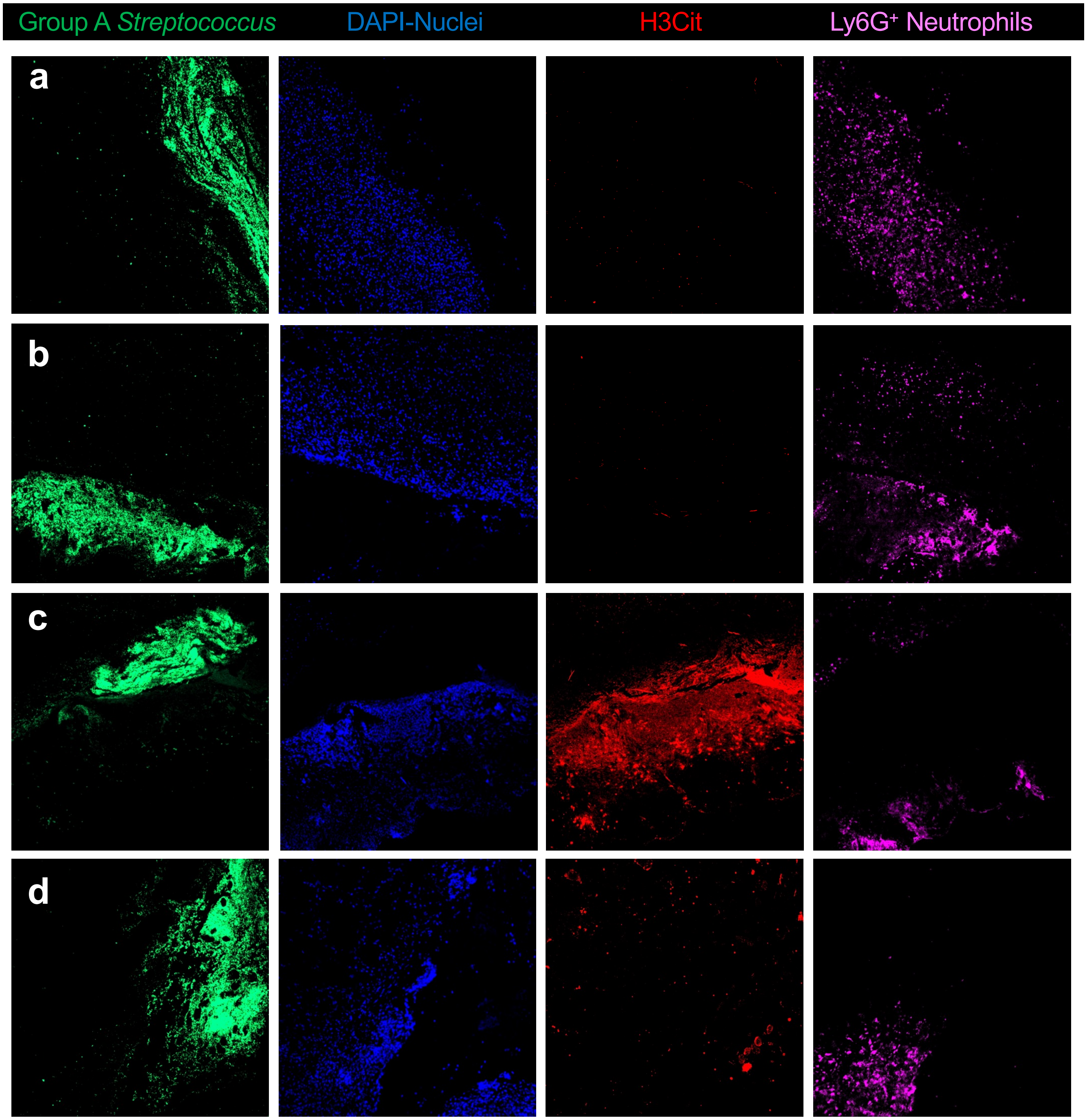

Supplementary Figure 10. Single channel images of H3Cit in infected skin tissue. C57BL/6J control (**a**, **b**) and STZ-induced diabetic (**c**, **d**) mice were subcutaneously infected with 10^7 CFU of either WT (**a**, **c**) or ΔClpX (**b**, **d**) *S. pyogenes*. Skin tissue was collected at 3 dpi, sectioned, and stained with FITC-conjugated anti-Group A *Streptococcus*, DAPI, Alexa Fluor 647–conjugated anti-Ly6G, rabbit anti-Histone H3 (citrullinated R2 + R8 + R17), and PE-conjugated goat anti-rabbit IgG secondary antibody to detect H3Cit.

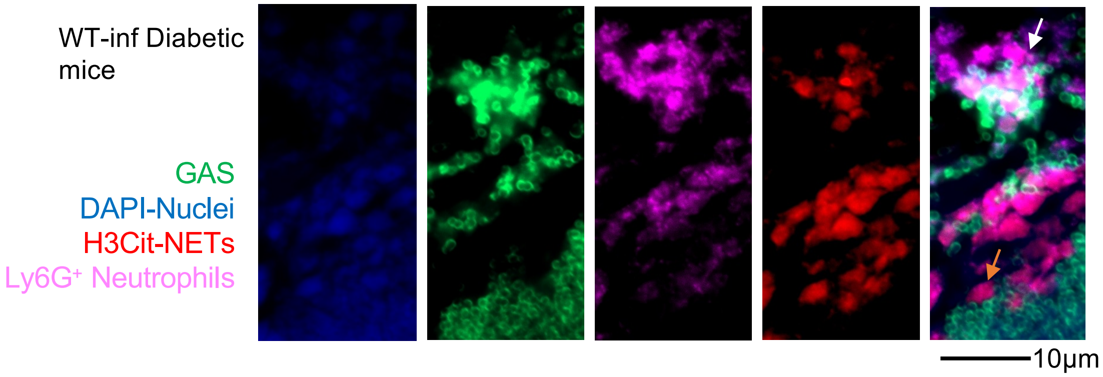

Supplementary Figure 11. Zoomed-in images of H3Cit in infected skin tissue. STZ-induced diabetic mice were subcutaneously infected with 10^7 CFU of WT *S. pyogenes*. Skin tissue was collected at 3 dpi, sectioned, and stained with FITC-conjugated anti-Group A *Streptococcus*, DAPI, Alexa Fluor 647–conjugated anti-Ly6G, rabbit anti-Histone H3 (citrullinated R2 + R8 + R17), and PE-conjugated goat anti-rabbit IgG secondary antibody to detect H3Cit.

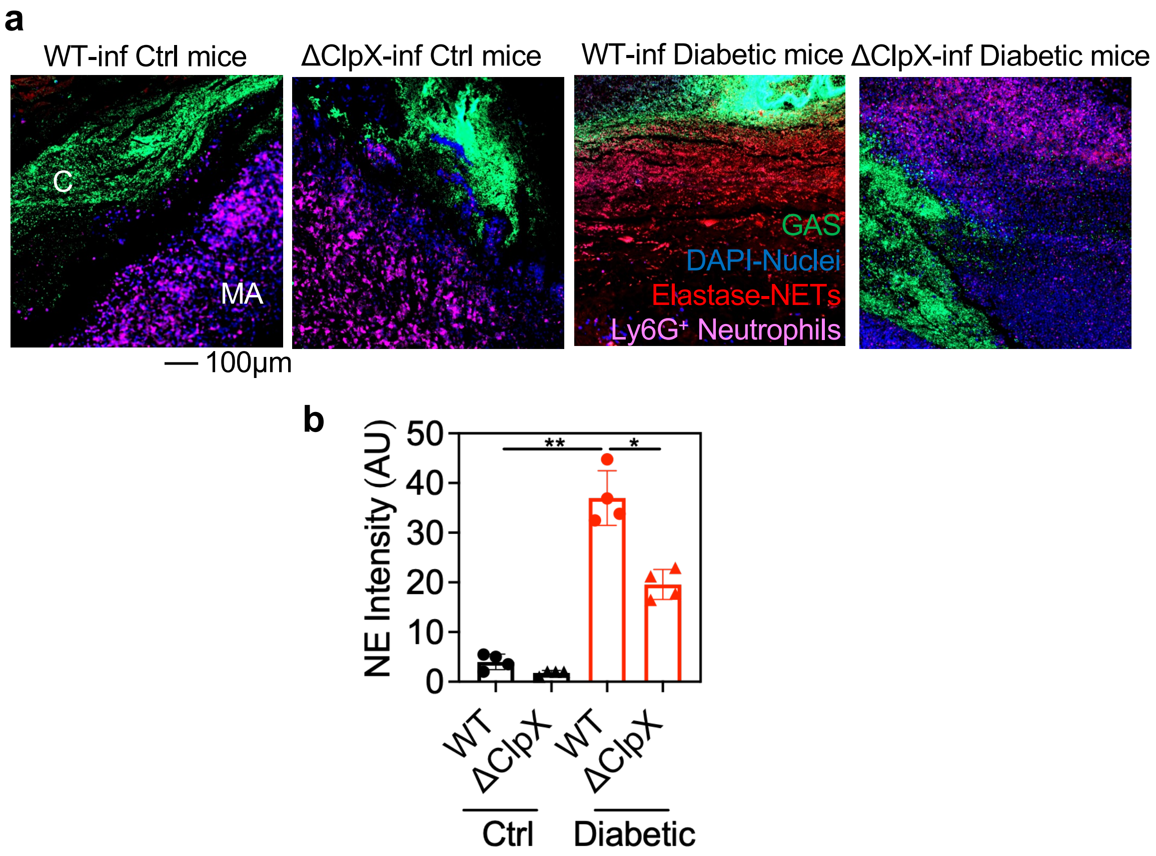

Supplementary Figure 12. Fluorescence microscopy of neutrophil elastase in infected skin tissue. C57BL/6J control and STZ-induced diabetic mice were subcutaneously infected with 10^7 CFU of either WT or ΔClpX *S. pyogenes*. (**a**) skin tissue was collected at 3 dpi, sectioned, and stained with FITC-conjugated anti-Group A *Streptococcus*, DAPI, Alexa Fluor 647–conjugated anti-Ly6G, and PE anti-neutrophil elastase (Bioss, bs-6982R-PE). (**b**) Quantification of NE intensity from tissue sections using ImageJ. AU: artificial unit. $p < 0.05$ (*), $p < 0.01$ (**), by two-way ANOVA with Tukey's post-hoc test.

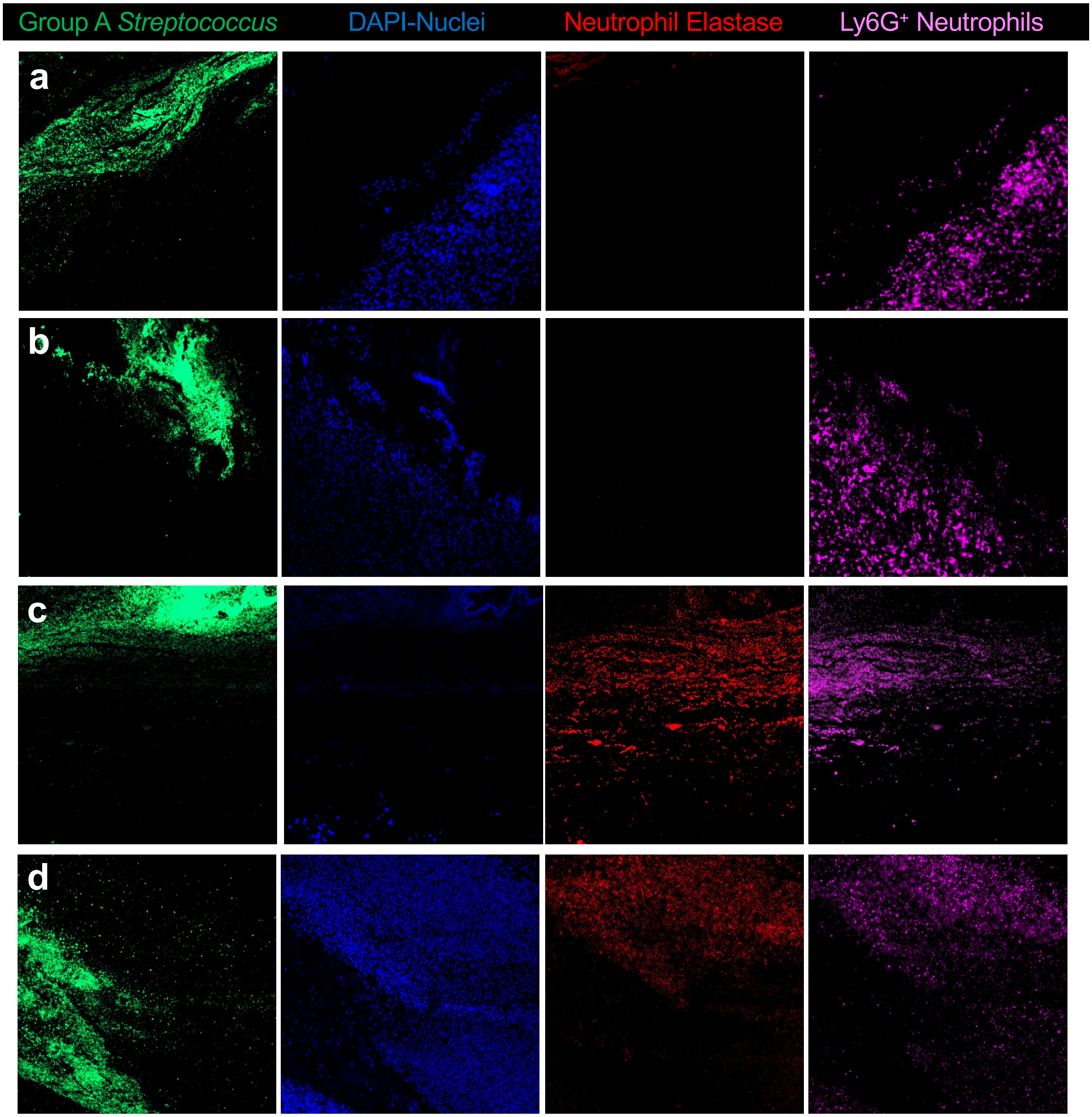

Supplementary Figure 13. Single channel images of NE in infected skin tissue. C57BL/6J control (**a**, **b**) and STZ-induced diabetic (**c**, **d**) mice were subcutaneously infected with 10^7 CFU of either WT (**a**, **c**) or ΔClpX (**b**, **d**) *S. pyogenes*. Skin tissue was collected at 3 dpi, sectioned, and stained with FITC-conjugated anti-Group A *Streptococcus*, DAPI, Alexa Fluor 647–conjugated anti-Ly6G, and PE anti-neutrophil elastase (Bioss, bs-6982R-PE).

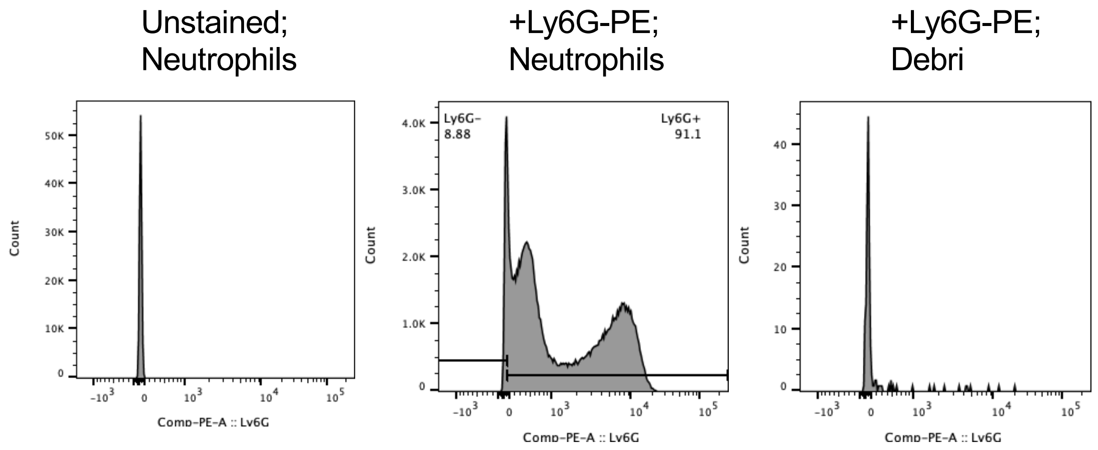

Supplementary Figure 14. Confirmation of neutrophil purity following Percoll gradient isolation. Bone marrow–derived neutrophils (BMDNs) were isolated from C57BL/6J mice using Percoll gradient. Following isolation, cells were stained with PE anti-Ly6G and analyzed by flow cytometry. Representative flow cytometry plots are shown. Neutrophils were defined as Ly6G^{+} cells, and purity typically exceeded 90%.

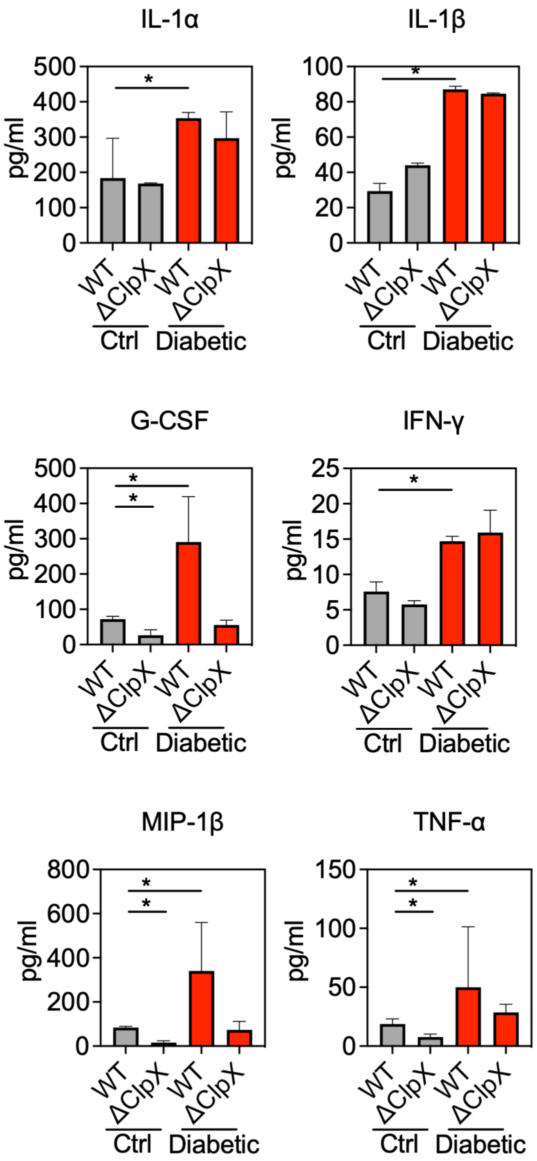

Supplementary Figure 15. ClpX promotes systemic inflammation in diabetic skin infection. C57BL/6J control and STZ-induced diabetic mice were subcutaneously infected with 10^7 CFU of WT or ΔClpX *S. pyogenes*. At 12 dpi, cytokine levels were measured in the supernatants of homogenized infected skin tissue. Data represent mean ± SEM from two independent experiments. $p < 0.05$ (*) by two-way ANOVA with Tukey's post-hoc test.
